# Adaptive Selection on Bracovirus Genomes Drives the Specialization of *Cotesia* Parasitoid Wasps

**DOI:** 10.1371/journal.pone.0064432

**Published:** 2013-05-28

**Authors:** Séverine Jancek, Annie Bézier, Philippe Gayral, Corentin Paillusson, Laure Kaiser, Stéphane Dupas, Bruno Pierre Le Ru, Valérie Barbe, Georges Periquet, Jean-Michel Drezen, Elisabeth A. Herniou

**Affiliations:** 1 Institut de Recherches sur la Biologie de l’Insecte, UMR 7261 CNRS, Université François-Rabelais, UFR Sciences et Techniques, Parc Grandmont, Tours, France; 2 Laboratoire Evolution, Génomes et Spéciation, CNRS UPR9034, IRD UR 072 and Université Paris Sud, Gif sur Yvette, France; 3 Unité de Recherche UMR 1272, Physiologie de l’Insecte, Signalisation et Communication, INRA, Versailles, France; 4 Icipe, IRD UR 072, Nairobi, Kenya; 5 Genoscope (CEA), CNRS UMR 8030, Université d'Evry, Evry, France; Onderstepoort Veterinary Institute, South Africa

## Abstract

The geographic mosaic of coevolution predicts parasite virulence should be locally adapted to the host community. *Cotesia* parasitoid wasps adapt to local lepidopteran species possibly through their symbiotic bracovirus. The virus, essential for the parasitism success, is at the heart of the complex coevolutionary relationship linking the wasps and their hosts. The large segmented genome contained in the virus particles encodes virulence genes involved in host immune and developmental suppression. Coevolutionary arms race should result in the positive selection of particular beneficial alleles. To understand the global role of bracoviruses in the local adaptation or specialization of parasitoid wasps to their hosts, we studied the molecular evolution of four bracoviruses associated with wasps of the genus *Cotesia,* including *C congregata, C vestalis* and new data and annotation on two ecologically differentiated populations of *C sesamie*, *Kitale* and *Mombasa.* Paired orthologs analyses revealed more genes under positive selection when comparing the two *C sesamiae* bracoviruses belonging to the same species, and more genes under strong evolutionary constraint between species. Furthermore branch-site evolutionary models showed that 17 genes, out of the 54 currently available shared by the four bracoviruses, harboured sites under positive selection including: the *histone H4-like*, a *C-type lectin*, two *ep1-like*, *ep2*, a *viral ankyrin*, *CrV1*, a *ben-domain*, a *Serine-rich*, and eight unknown genes. Lastly the phylogenetic analyses of the *histone, ep2* and *CrV1* genes in different African *C sesamiae* populations showed that each gene described differently the individual relationships. In particular we found recombination had happened between the *ep2* and *CrV1* genes, which are localized 37.5 kb apart on the wasp chromosomes. Involved in multidirectional coevolutionary interactions, *C sesamiae* wasps rely on different bracovirus mediated molecular pathways to overcome local host resistance.

## Introduction

How populations and species evolve *in situ*? A major challenge in biology is to capture evolution as it happens in the field, especially both at genetic and ecological scales. The field of evolutionary and ecological functional genomics aims to unravel the genetic bases of adaptation to the environment [1]. A critical step in assessing the impact of adaptation on complex genomes is the definition of ecological niches. Strongly constrained by their hosts, the niche of obligatory parasites can easily be identified during sampling even if it can change over time. In the frame of the geographic mosaic of coevolution, the host community is predicted to vary geographically and to adapt locally in response to parasite virulence traits [2]. Antagonistic coevolution between hosts and parasites thus results in complex evolutionary arms race, which can be associated with rapid adaptive changes [3], [4], [5], in accordance with the Red Queen theory [6]. At the molecular level, the consequences of coevolutionary arms race should be the selection of particular beneficial alleles and accelerated mutation accumulation for example in duplicated genes creating different weapons for more host targets [7], [8].

Polydnaviruses (PDVs) are unusual viruses integrated into the genome of parasitic Hymenoptera. Wasps have domesticated PDVs to deliver molecular weapons to fight the immunity of their lepidopteran hosts [9], [10]. Thus PDVs are at the heart of complex co-evolutionary interactions, both as pathogens of the parasitized moths and as mutualists of parasitoid wasps [11]. In obligate symbiotic associations with some subfamilies of Braconidae and Ichneumonidae, PDVs are classified into the *Bracovirus* (BV) and *Ichnovirus* (IV) genera respectively [12]. If bracoviruses originated from the capture of a nudivirus 103 Mya [13], [14], ichnoviruses derived from a different as yet uncharacterized virus by convergent evolution [15]. Two gene families coding for virulence factors are conserved between the available bracovirus and ichnovirus genomes [16], [17], outlining the potential adaptive role of PDVs for parasitoid wasps.

PDV genomes exist in two forms: a provirus in the form of multiple segments permanently integrated into the wasp genome, which mediates PDV vertical transmission [9], [18], and a free form composed of several double stranded DNA circles, packaged in viral particles [19], [20]. These particles are only produced in specialized cells of the wasp ovaries [21], [22]. They are injected into lepidopteran hosts during wasp oviposition. The gene content of the PDV packaged genomes varies depending on the wasp lineage from 60 to 260 genes [11], [18], [20], [22]. These genes expressed in infected caterpillar tissues produce virulence factors manipulating host physiology and altering host immunity or development [23], [24]. They are indispensable for the parasitic wasp success [25], [26], [27]. Phylogenetic analyses of bracoviral genes encoding sugar transporters showed these PDV genes clearly had a wasp origin [8]. However for most PDV genes, nucleotide divergence is such that phylogenetic links with wasp genes are no longer detectable.

Comparative studies suggest that PDV genome contents are linked with wasp phylogenetic history, as well as correlated with specific host/parasite interactions [8], [21]. The large number of virulence genes encoded in PDV genomes illustrates the complex relationships linking PDVs, wasps and lepidopteran hosts [21], [22]. In the braconid *Cotesia sesamiae,* there is a correlation between different allelic forms of the virulence gene *CrV1* and parasitic success in particular host species. This suggests adaptive PDV genotypes have been selected for the suppression of local host resistance [7], [28].

We aim to understand the role of PDVs in the adaptation and/or specialization of parasitoid wasps to particular host panel. We focused on the bracovirus genomes of parasitic wasps belonging to the genus *Cotesia* (Braconidae): *C. congregata* (Say), *C. vestalis* (Kurdjumov; synonym to *C. plutellae*) and *C. sesamiae* (Cameron). If *C. congregata* has been described to parasitize 14 Sphingidae species belonging to different genera [29], [30], [31], the population studied has been reared for many generations only on *Manduca sexta* L. (Sphingidae). Similarly if *C. vestalis* has been reared from several species belonging to different families [32], [33], the population studied comes from a lab colony reared on *Plutella xylostella* L. (Plutellidae). As for *C. sesamiae*, this wasp parasitizes over twenty species of African stem borer belonging to the Noctuidae and Crambidae families [7]. Here we study genotypes from two ecologically differentiated wild populations, *Kitale* and *Mombasa*, with different host preferences but retaining a generalist status [7], [34]. To investigate the genetic bases of wasp adaptation, we compared the molecular evolution and positive selection of all orthologous BV genes both between and within wasp species and further investigated the adaptive role of 3 genes in 21 wild *C. sesamiae* populations.

## Material and Methods

### *Cotesia sesamiae kitale* Bracovirus (CskBV) Genome Sequencing

The ovaries of 20 *C. sesamiae kitale* wasps (ICIPE laboratory strain) were dissected in TE buffer. CskBV particles were purified by filtration on SPinX columns (Corning). Encapsulated viral DNA was then extracted using the QiaAmp DNA extration Kit (Qiagen) and amplified using the rolling circle TempliPhi DNA polymerase (Amersham Biosciences). CskBV DNA library was prepared with GS FLX Titanium Rapid Library Preparation Kit and sequenced using a 454 GS-FLX Titanium system (1/8 run) [35]. The 454 raw data is deposited to the EMBL Sequence Read Archive under the accession ERR229552.

### Assembly and Annotation of CskBV and CsmBV

CskBV 454-reads were assembled with Newbler *de novo* Assembler software 2.5.1 (Roche454) [35]. *Cotesia sesamiae mombasa* (Csm) and *Cotesia sesamiae kitale* (Csk) BAC sequences (EF710626-43) corresponding to partial sequences of the proviral locus were retrieved from the NCBI portal and assembled with Geneious assembly software [36]. Contigs corresponding to viral circles were identified by sequence homology with CcBV and CvBV segments retrieved from GenBank (accession numbers CcBV: AJ632304-33, and FR873483-87; CvBV: HQ009524-58).

Gene predictions on CskBV newly sequenced contigs and BACs and on CsmBV BACs were made with SoftBerry’s FGENESH *de novo* prediction tool [37] and FGENESH+ using the honeybee (*Apis mellifera* L.) training set and were manually corrected by comparison with known bracoviral genes. Genes that displayed high similarity with insect genes in GenBank or with *C. congregata* wasp genes were considered to belong to wasp genome and were excluded from our analyses. Gene models from FGENESH were generally accepted unless experimental information contradicted those models.

CskBV contigs corresponding to PDV genome segments were used to confirm the proviral form on the BACs derived from the wasp genomes. This allowed the recognition of conserved DRJ motif (Direct Repeat Junction) allowing the excision of viral segment from the wasp genome [38], [39], [40]. CskBV 454-data experimentally demonstrate the production of viral segments from proviral sequences. When no DRJ was found, in particular at the start/end of BACs inserts, genes from these zones were compared by BLAST to GenBank database and with the integrated form of CcBV genome [18] to determine whether they corresponded to partial proviral segments.

Wolf PSORT 0.2 was used to predict protein subcellular localization [41]. Localization was considered to be correctly inferred when the first localization score was equal or superior to seven and the second localization, if present, was at least inferior to half the first score.

### Molecular Evolution of Orthologous Gene

We used Exonerate 2.2.0 [42] to assess orthologous pairs of genes based on reciprocal best hits, between each pair of viruses (CskBV/CsmBV, CcBV/CvBV, CcBV/CskBV, CcBV/CsmBV, CvBV/CskBV and CvBV/CskBV). Two genes were considered orthologous when the average amino acid similarity was over 70% for 95% of their length. In the case of gene families containing unequal copy numbers in different genomes, conserved genome synteny was used to infer orthology. Likewise gene order conservation was used to confirm the orthology relationships for the *ep2* gene, which had only 60% similarity between CsBV and CcBV. Furthermore logical transitivity relationships between the four genomes were applied to make sure the genes were called to the same groups in all pairwise analyses. If ever uncertainties remained, we preferred calling similar genes as belonging to different orthologous groups. The orthologous genes present in all genomes derive from these analyses.

Each pair of orthologs was aligned with ClustalW 2.0 [43], [44] with default parameters allowing the estimation of average amino acid identity. To ensure that all alignments were suitable for evolutionary analyzes, we tested for the presence of substitution saturation using the DAMBE software [45]. Phylogenetic information is lost when the observed saturation index is greater than or equals to half the full substitution saturation (expected saturation indices). Saturation can be detected when the observed indices are higher than the expected indices [46]. Pairwise estimates of the synonymous (dS) and non-synonymous (dN) substitution rates were obtained from PAML 4.4 (runmode -2) with default parameters. Statistical significance of differences between dN/dS mean values of all comparisons was tested by one-way ANOVA and post hoc pairwise comparisons were done by Tukey's HSD (Honestly Significant Difference) test, using the PAST software [47].

From the pairwise analyses described above, we identified a subset of orthologous genes shared between the four BVs. Alignments of each common ortholog were performed with Mafft 6.8.11 [48] and were then submitted to BMGE 1.0 [49] to eliminate poorly aligned positions and divergent regions that could be misleading for dN/dS analyses. The dN/dS ratios were first calculated using the FEL model in HYPHY 2.0 [50]. We estimated the best model for each common gene with modeltest.bf [51]. A Likelihood ratio tests (LRT) was used to assess the significance of positively selected codons (*p*-value threshold = 0.01). We then performed site model analysis by CODEML, implemented in PAML v4.4 [52] using simple and complex models: M1a (NSsites = 1), M2a (NSsites = 2), and M8 (NSsites = 8; omega estimated) and M8a (NSsites = 8; fix_omega = 1). LRT [53], [54] were performed to compare models M1a-M2a and M8-M8a with type I error = 0.01 and df = 2 and df = 1 [55], respectively. When the test was significant (*p*-value<0.01), Bayes Empirical Bayes (BEB) inference was used to identify amino acid sites under positive selection [56]. To eliminate false-positive detection, results of both PAML (M1a-M2a and M8-M8a) and HYPHY were compared. We only kept the result when the same genes and same amino acid were detected under positive selection. Non-synonymous substitutions driven by positive selection were then classified as ‘radical’ or ‘conservative’, denoting whether they involved change in amino acid physico-chemical property, such as charge, volume or polarity (as proposed by Zhang [57] and Hughes *et al* [58]). Structure and function prediction of the positively selected codons were performed with profile hidden Markov model (HMM-HMM) comparison using HHPreds software [59] on Protein DataBase PDB-70_6Dec12 [60]. Multiple sequence alignment generations were performed by HHblits program [61] using default parameters. Hits showing a probability >95% were retained as significant. The best hit was analyzed further. Independence between the selective pressure on 54 orthologous genes (positive vs. neutral or negative) and PDV circles was tested with a Fisher exact test using R software [62].

### Bracovirus Evolution within *Cotesia sesamiae*

We sequenced the *viral histone H4-like*, *ep2* and *CrV1* genes of additional genotypes from 17 *Cotesia sesamiae* populations. The wasps were collected in the field between 2004 and 2010 in Kenya, Eritrea, South Africa, Mozambique, Tanzania, Democratic Republic of Congo (DRC) and Cameroon (Table S1; [7]). Each genotype was sequenced from a cocoon mass reared from a single host caterpillar, identified as belonging to 14 species from 6 genera (Table S1). Each cocoon mass is assumed to be the progeny of a single female. Samples were stored in 95% ethanol and DNA extracted from a single wasp. *Cotesia* bracovirus specific primers were used for PCR amplifications and sequencing (histone: HistCsBV-F 5′-ATGTCTGATTGTCCTAAAGAT-3′ and HistCsBV-R 5′-TCAACCTCCATAACCATAGAT-3′; EP2: EP2Cs-F 5′-CTAAGCAGAAGAACTTCTTC-3′ and EP2Cs-R2 5′-TCAGTTGCGTTTAACTCG-3′; see [28] for *CrV1*). Sequences were aligned using MAFFT with default parameters in Geneious package and the positions of indels manually edited to maximize nucleotide identity [36]. Population clustering trees were obtained using HKY distance neighbor joining in PAUP and 1000 bootstrap replicates were performed to assess robustness [63]. We further performed Branch-site REL analyses in HYPHY to detect branches showing signs of positive or purifying selection [64].

## Results and Discussion

### CskBV Genome Sequence and *Cotesia bracovirus* Comparative Genomics

**Figure 1:**
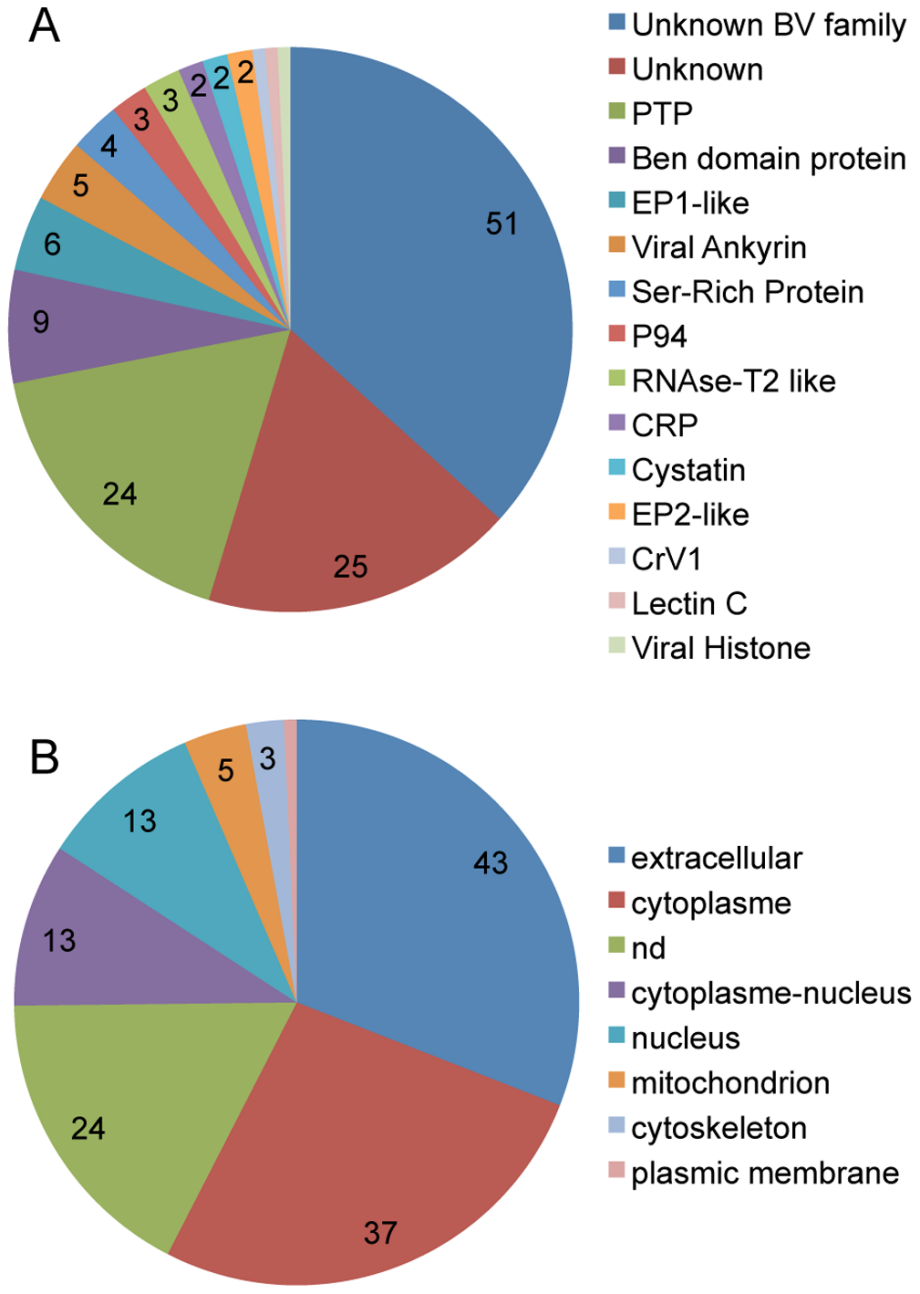
Functional analysis of 139 CskBV genes. (A) Bracovirus gene homologies identified by BLAST; (B) PSORT protein sub-localization analysis; within the pie charts values indicate the number (>1) of genes identified in each class; nd: not determined.

**Figure 2:**
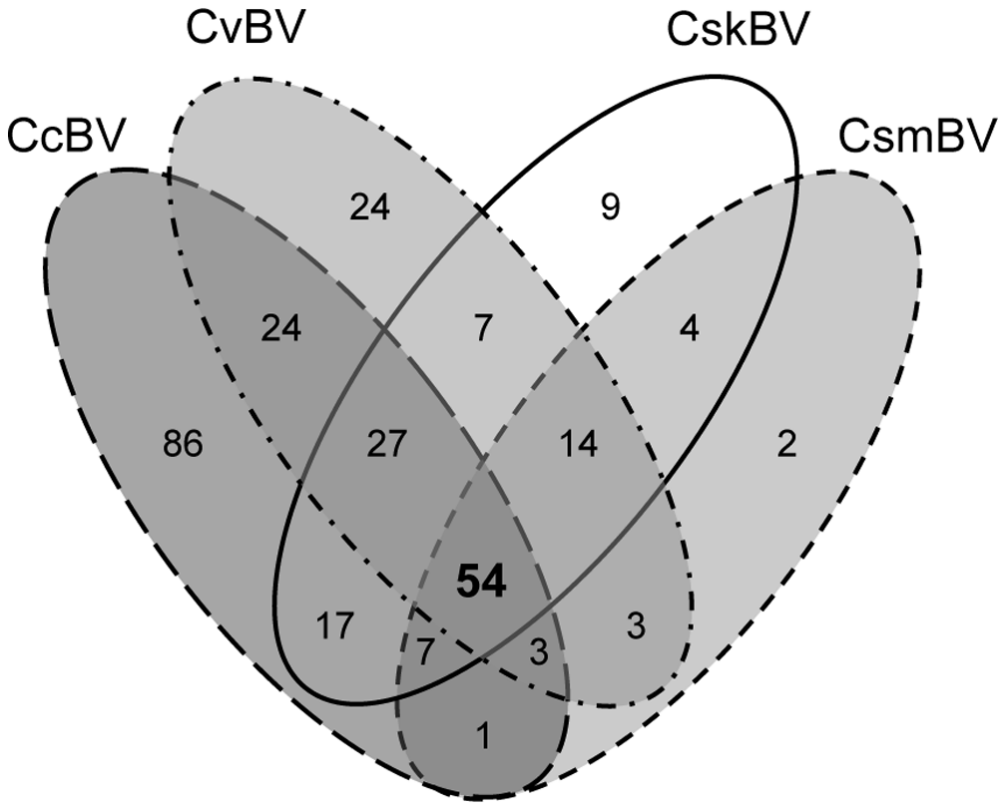
Gene content similarities between 4 *Cotesia* bracovirus genomes. Values within the Venn diagram indicate the number of genes identified in each class; orthologs were identified by reciprocal best hits.

Given the segmental nature of bracovirus genomes packaged in the particles (i.e. multiple DNA circles each encoding few genes) particular effort had to be made to assess the completeness of the data available for the four *Cotesia* wasps (Table 1). With 260 predicted genes for 35 segments and 158 genes for 35 segments respectively, the CcBV and CvBV genomes were considered complete [18], [20], [65]. Despite having the same number of segments, the use of different annotation methods on CcBV and CvBV genomes, such as the inclusion of CDS encoding proteins smaller than 100 AA, could explain the difference in total gene number (Table 1). Both *C. sesamiae* bracoviruses were considered incomplete by ∼1/3 for CskBV and by >1/2 for CsmBV, since their complete genome is likely similar in size and structure to CcBV and CvBV (Table 1). Following the assembly and annotation of *C. sesamiae kitale bracovirus* (CskBV), we found 139 genes (Table 1; Table S2; GenBank accessions HF562906-31 and HS570923-29); 131 of those genes were encoded on 26 circles (16 complete and 10 incomplete circles) and 8 were identified from isolated contigs. Bracovirus homologues were found for 126 genes (Figures 1A, 2), as could be expected given the phylogenetic proximity of *C. sesamiae bracovirus* to those of *C. congregata* and *C. vestalis.* They could be classified either as single gene orthologs or as belonging to bracovirus gene families. Only 15 genes appeared to be specific to CsBV, against 86 in CcBV and 24 in CvBV (Figure 2).

**Table 1:** *Cotesia* bracovirus genome features.

Feature	CcBV	CvBV	CskBV*	CsmBV*
Estimated genome size	720 Kb	540 Kb	373 Kb	271 Kb
Number of circles	35	35	26	16
G+C content	34%	34.4%	34.1%	39%
Predicted genes	260	158	139	88
Coding density	22%	27%	27%	24%

* Incomplete genomes: the current size estimate corresponds to BAC sequences and to additional 454 sequences for CskBV.

Subcellular localizations of 115 CskBV proteins (Figure 1B) could be predicted using Wolf PSORT analyses [66]: 31% CskBV protein appeared to be addressed to the extracellular compartment, such as EP-like proteins, and 27% to the cytoplasm, including some PTPs. However two PTPs (PTP-*thau* and PTP-*alpha*) had a nuclear sublocalization signals indicating potential activity in the nucleus. As cellular PTPs are involved in signal transduction [67] and as bracovirus PTPs are supposed to disrupt host signal transduction pathways [68], [69], this suggests that these 2 PTPs could also target some late steps of signal transduction in the nucleus [69]. A sublocalization of the PDV histone H4-like was also predicted in the nucleus, consistent with its role in altering the expression of several host genes [70], [71] and with the localization of cellular histones.

Concerning the BV of the *C. sesamiae mombasa* strain (CsmBV), the annotation derived from BAC sequences predicted 97 genes distributed on 16 segments, corresponding to 14 complete and 2 partial segments (Table S3; Genbank accessions EF710636-43). Of note, *cystatin* genes were not present in the annotated BAC from CsmBV, and only 10 *ptp* genes were found out of the ∼30 copies expected for this gene family [72]. Only two genes (of unknown function) had not been identified in the other *Cotesia* BV (Figure 2). The higher 38% GC content for CsmBV genome in comparison to the 34% for other *Cotesia* BVs (Table 1) could be explained by a bias resulting from its incompleteness.

Like it has been shown for the *CrV1* gene [73], we observed a strict co-phylogeny between whole bracovirus genomes (based on global amino acid identity) and their carrying wasps species (Figure S1 in File S1).

### Paired Orthologs Analyses

To assess the effective implication of bracovirus genes in the ecological adaptation of *Cotesia* wasps, we measured the rate of non-synonymous versus synonymous substitutions, as more amino acid changes are expected in genes involved in adaptation [52]. We identified 65 to 108 orthologs for each bracovirus pair (Figure 2). For example, between CcBV and CvBV, we found 108 orthologs corresponding to 48% and 68% of CcBV and CvBV genome contents respectively (Table S4). With 86 unique genes, CcBV had the largest number of private genes (Figure 2). Saturation analyzes, on the alignments of the 54 genes shared by the four Cotesia-BV and of the 110 genes from the two most distantly related species CskBV-CcBV, did not reveal any sign of substitution saturation (p<10^−4^, Table S5 and S6), thereby validating our dataset for phylogenetic analyses and dN/dS estimations [45], [46].

**Figure 3:**
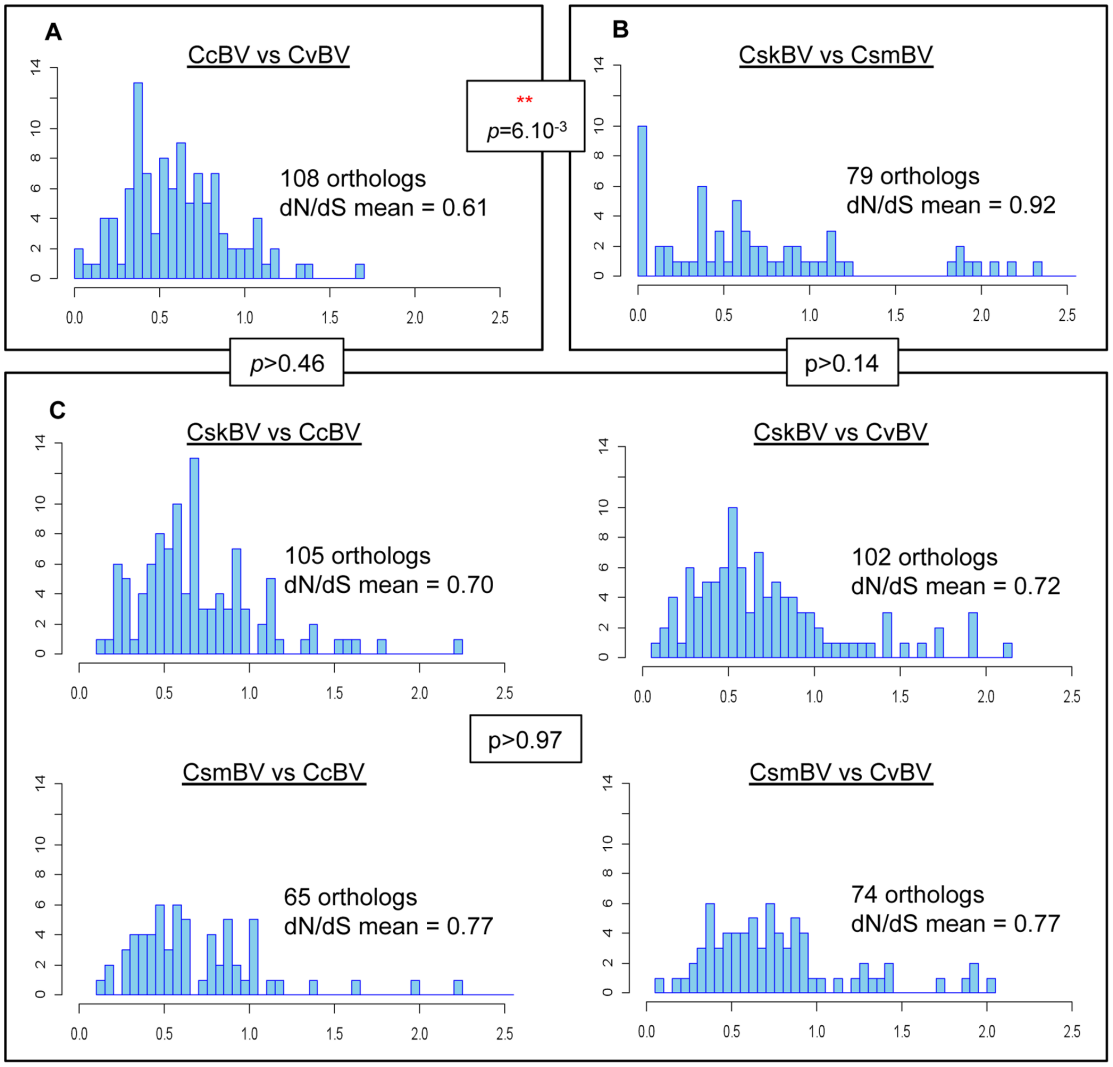
Pairwise dN/dS ratio distribution in *Cotesia* bracovirus orthologous genes. (A) interspecific comparisons CcBV vs CvBV; (B) Intraspecific comparison CskBV vs CsmBV; due to weak polymorphism of several orthologs identified between CskBV and CsmBV, only 66 dN/dS ratios could be correctly estimated with codeml (PAML) among the 79 orthologous pairs; (C) CcBV vs CskBV, CcBV vs CsmBV, CvBV vs. CskBV and CvBV vs CsmBV. The x axis represents dN/dS ratio and the y axis the number of orthologs found 0.05 dN/dS intervals.

Pairwise dN/dS analyses on orthologous genes were performed interspecies (CcBV, CvBV and CsBVs) and intraspecies (CskBV and CsmBV), (Figure 3). Theoretically dN/dS ratios are expected to be statistically higher between species than within species [74], [75]. When comparing all pairwise analyses, we observed significant differences in dN/dS ratio distribution profiles (ANOVA, *F* = 2.612, *p* = 0.024). Surprisingly, we found dN/dS ratios were significantly higher in interspecific than in intraspecific comparisons (HSD test, *Q* = 4.95, *p* = 0.006). The lowest dN/dS mean value (0.61) was found for the CcBV/CvBV ‘interspecific’ comparison, (Figure 3A). Forty-three genes (38%) were under strong purifying selection (dN/dS<0.5) in the CcBV/CvBV comparison. This reflects a global evolutionary constraint to conserve specific gene functions since the diversification of the two species. In contrast, we found the highest dN/dS for the CskBV/CsmBV ‘intraspecific’ comparison, showing a mean value of 0.92 and a scattered distribution profile (Figure 3B). When comparing genes, there were 28 orthologs (35%) under strong evolutionary constraint. We found 12 genes (15.2%) under positive selection (dN/dS>1,8), while none reached this value in the CcBV/CvBV comparison. This high number of positively selected genes could reflect the ongoing ecological selection of new or better-adapted protein functions [76]. It therefore appears seemingly contrary to predictions [74], [75], that during *Cotesia* bracoviruses speciation, strong purifying forces follow a first period of positive selection in most genes.

The number of orthologs under relaxed constraint (dN/dS around 1) is quite constant and abundant for the different interspecific comparisons (∼60%). This could be explained by the presence of large multigenic families such as the *ptp* or *ankyrin* gene families, resulting from numerous gene duplications [77]. Under the hypothesis of evolution by gene duplication, functional redundancy of the duplicated pair can trigger two distinct fates. The duplicated copy could undergo relaxed selective pressure, allowing both copies to share the same initial functions (sub-functionalization). Alternatively, new specific constraints can appear in the duplicated copy leading to the apparition of entirely new functions (neo-functionalization) [17], [78], [79]. Comparison of CsBV genotypes (CsmBV or CskBV) vs CcBV or CvBV (Figure 3C) showed an intermediate profile with around 35% genes under strong selection pressure but also around 9% genes under positive selection pressure. Mean dN/dS ratios did not differ significantly among other pairwise comparisons (HSD tests, *p*>0.08). We obtained similar results when restricting the dN/dS pairwise analyses to the 54 orthologs common to all 4 wasps (data not shown), showing incomplete gene sampling was not biasing our results.

The contrasted evolutionary rates we observed at the within and between species levels show the overall adaptive role of bracovirus genes in wasp local adaptation. Positive selection pressure could drive the evolution of new genes or new functions better adapted to new or predominant niches, different lepidopteran host species in our case. This could lead to the specialization of particular wasp population towards particular lepidopteran host species [7], [28], [80]. Thus bracovirus mediated speciation could occur for wasp populations living in different environments in response to the abundance of particular host species.

### Common Orthologous Genes Analyses

To take into account the phylogenetic history of the genes, we analyzed the molecular evolution in all orthologous genes shared by the four *Cotesia* bracoviruses. To assemble this smaller subset, we considered any gene with ortholog in the other three bracoviruses. We first found a set of 54 orthologs shared by CcBV, CvBV, CskBV and CsmBV (Table S7), which represented from 24% (CcBV) to 61% (CsmBV) of the characterized gene contents. This low number of common genes seemed to be linked with the fact that the number of paired orthologs was underestimated due to the incomplete genome data and divergence in segment duplication history between bracoviruses [18], [81]. Hence, we also performed the analyses with a second set of 81 genes obtained by removing CsmBV, the most incomplete genome.

BLAST homology searches indicated that only 24 of the 54 common genes (44%) encoded proteins showing significant similarity to members of previously identified bracoviruses gene/protein families. Similarly, 39 out of 81 genes were found when analyzing the genes in common between CskBV, CcBV and CvBV. Most of the core of orthologs (56%) had unknown functions. Among identified functions, we found eight protein tyrosine phosphatases (PTP), the viral histone H4-like, a C-type lectin, three viral ankyrin, three EP-like proteins, two P94-like proteins, a CRP3-like, the CrV1 protein and two Ser-Rich proteins (Table S7). Most of these proteins seem implicated in the disruption of host immune response and/or host development [28], [82], [83], [84].

We analyzed the molecular evolution of the common orthologous genes in a phylogenetic context by estimating the dN/dS ratio with HYPHY and PAML [50], [52]. We found seventeen proteins presenting sites under positive selection: the histone H4-like, a C-type lectin, two EP1-like proteins, an EP2, a viral ankyrin, CrV1, a ben-domain protein, a Ser-Rich protein, and eight unknown proteins (Table 2). In all 17 proteins we found positively selected sites accompanied by ‘radical’ rather than ‘conservative’ changes in the physico-chemical property of the amino acid (Table 2) [57]. In the absence of further functional evidence, this reinforces our conclusions that the substitutions in these genes could be adaptive, under the assumption that radical replacements are more likely than conservative replacements to improve the function of a protein [57], [58]. Overall the relatively high number of positively selected bracovirus genes, clustered as a proviruses in the wasp genome, likely reflects the genuine high adaptive nature of PDVs rather than artifactual results given all the precautions taken to discard false positive sites.

**Table 2:** Detection of amino-acids evolving under positive selection.

Gene^a^	Function	Positivelyselected sites^b^	dN/dS estimation(+/− SE) c	Positive selectionProbability^d^	AA changes	AA Biochemicalcategory^e^
ank-8 CskBV_16.2	viral ankyrin	100	7.525+/−2.605	0.976	Y/T/F	2 2 1
Ben CskBV_27.3	ben-domain protein	626	9.215+/−1.665	0.983	E/A/G	4 1 2
bv11 CskBV_36.5	unknown bv11 protein	2	9.597+/−2.518	0.983	M/G/W	1 2 1
		89	6.643+/−2.476	0.991	W/K/V	1 2 1
bv15 CskBV_2.4	unknown bv15 protein	3	5.310+/−2.090	0.981	P/C/F	1 2 1
		40	5.295+/−2.108	0.977	L/V/Q	1 1 2
		51	5.291+/−2.101	0.978	E/M/D	4 1 4
		76	5.279+/−2.109	0.976	V/Q/K	1 2 3
bv2 CskBV_2.5	unknown bv2 protein	216	4.724+/−1.454	0.978	I/P/V	**1 1 1**
		218	4.794+/−1.365	0.996	C/G/R/H	2 2 3 3
bv5 CskBV_33.1	unknown bv5 protein	107	5.582+/−1.674	0.980	Q/E/I	2 4 1
		124	5.664+/−1.573	0.996	M/L/H	1 1 3
		127	5.670+/−1.565	0.997	G/P	2 1
		128	5.663+/−1.573	0.995	S/E/D	2 4 4
bv6 CskBV_32.14	unknown bv6 protein	92	10.101+/−0.832	0.997	Y/E/I	2 4 1
		98	10.105+/−0.810	0.997	C/T/K	2 2 3
		100	10.119+/−0.723	0.999	I/Q/T	1 2 2
		104	10.089+/−0.898	0.996	S/N	**2 2**
		107	10.124+/−0.690	1.000	T/R/A	2 3 1
		110	10.125+/−0.685	1.000	P/S/Y	1 2 2
CcBV_18.13-like CskBV_18.1	unknown	129	9.569+/−1.368	0.992	N/S	**2 2**
		140	9.340+/−1.946	0.967	I/G/V	1 2 1
CcBV_24.2-likeCskBV_24.4	unknown	63	2.957+/−0.846	0.980	S/D/L	2 4 1
crv1 CskBV_13.5	CRV1	91	3.810+/−1.678	0.984	E/K/G/M	4 3 2 1
CskBV_2.7	unknown	286	3.186+/−1.041	0.996	W/Y/D	1 2 4
ep1-like CskBV_5.4	EP1-like	25	2.548+/−0.440	0.984	N/Y/Q/E	2 2 2 4
		73	2.552+/−0.435	0.986	T/N/S/E	2 2 2 4
		109	2.549+/−0.440	0.984	V/N/R/	1 2 3
ep1-like CskBV_37.1	EP1-like	18	5.469+/−1.559	0.992	R/G/L	3 2 1
		39	5.466+/−1.560	0.992	D/H/T	4 3 2
		104	5.407+/−1.633	0.980	M/S	1 2
		244	5.422+/−1.614	0.983	L/I/T	1 1 2
ep2 CskBV_2.1	EP2	280	2.840+/−0.728	0.985	M/H/T	1 3 2
Histone CskBV_7.1	Viral Histone H4-like	2	9.009+/−1.471	0.996	S/A/I	2 1 1
		11	8.884+/−1.770	0.981	E/V/G	4 1 2
		21	9.031+/−1.413	0.998	F/L/Q	1 1 2
		38	8.868+/−1.805	0.979	S/A/G	2 1 2
		96	9.022+/−1.436	0.997	Q/I/H	2 1 3
Lectin CskBV_13.9	Lectin-C	85	6.649+/−2.331	0.997	R/G/L/T	3 2 1 2
ser-rich6-likeCskBV_18.2	Ser-Rich protein	11	6.986+/−2.429	0.984	S/P/F	2 1 1
		23	6.960+/−2.453	0.980	L/A/M	**1 1 1**

a CskBV gene name based on CcBV homology.

b Codon position refers to CskBV sequence.

c Estimates are from models M8 and M8a (PAML).

d Posterior probability from BEB inference (Type I error = 5%), Model M8 (PAML).

e Non-polar R groups: 1, Polar R groups: 2, Positively charged R groups: 3, Negatively charged R groups: 4. Substitutions not changing biochemical properties are indicated in **bold**.

Given the high number of PDV genes of unknown functions and the relative scarcity of PDV functional studies, we can only infer very hypotheses as to the particular role of the genes we found under positive selection. The histone H4-like, having strong homology with insect histones (including the host), had been reported to have an immunosuppressive effect on hemocyte spreading of lepidopteran host by altering expression of several genes possibly inducing epigenetic transcription control [70], [85].

Finding a *lectin* among the common orthologs of the four Cotesia bracoviruses is interesting, as C-type lectins bind to specific glyco-residues on the surface of damaged tissues or foreign bodies, inducing immune response [86], [87]. This potentially represents a novel approach used by bracoviruses to alter immune responses in parasitized hosts [88]. Bracoviral lectins might be competing with lepidopteran host lectins for binding sites involved in the induction of immune reactions [86], [87]. In that case, this suggests parasitoid larvae escape from host immune defenses could also involve an interference with their host non-self recognition.

The *ep* genes have so far only been found in bracoviruses [20], [22], [86]. The high and fast expression levels of EP-like proteins (EPs) in parasitized host suggest immunosuppressant functions acting early after oviposition [89], [90] possibly through hemolytic activity [91]. Moreover, there is a positive correlation between the quantitative level of EP gene expression and the host tolerance to the parasitoid larvae [83]. Together with our molecular evolution results, this suggests that the adaptive role of EPs is based both on quantitative expression levels correlated with variation in host tolerance, and on the qualitative level, positive selection acting on the primary sequence specificity to particular host immune factors.

Concerning the other genes, the P94-like and the CRP3-like proteins were under strong evolutionary constraint. The PTPs were under global relaxed constraint. This is consistent with the fact that we found eight different PTPs among the common orthologous genes of the 4 bracoviruses. Indeed, the *ptp* genes are numerous in several bracovirus genomes [8], [11], [20]. This suggests that duplication of PTP sequences has been recurrent and that duplicated PTP have been the subject of sub-functionalization with conservation of the catalytic site during the evolution of the bracovirus genomes. The relaxed selection pressure on particular *ptp* genes might be linked with the instrumental role of PTPs for successful parasitism [68]. Furthermore, as each PTP has a specific substrate [68], [72], bracovirus PTP diversity could reflect their potential capacity to interact with different molecular targets in their different lepidopteran hosts [69], [72]. This indicates the potential implication of the *ptp* gene family in host range definition.

To evaluate further the functional importance of positively selected codons, biochemical features of the proteins were analyzed using profile hidden Markov model homology searches. Significant homology and biochemical information based on PDB database could be assessed for four genes. The Histone (CskBV_7.1) was found to be homologous to protein Histone H4 (Uniprot P22799). The positively selected residue 38 is located 3 AA before the DNA binding domain, and residue 96 inside the domain core histone H2A/H2B/H3/H4. For the lectin (CskBV_13.9), the residue 85 was found to be located in the Lectin C-type domain of protein C-type Lectin domain family 9 member A (Uniprot Q6UXN8). The EP1-like (CskBV_5.4) protein harbors a domain Pectate Lyase C of the homologous protein Pectate trisaccharide lyase (Uniprot Q9WYR4). Both 117 and 165 positively selected residues belong to domain Pectate Lysae C and residue 117 is located only14 AA upstream of the active site. For EP1-like (CskBV_37.1), residues 18 and 39 were also located in the domain Pectate Lyase 3 of the homologous protein Poly(beta_D_mannuronate)_C5 epimerase 4 (Uniprot Q44493). Altogether these results show that the positively selected sites we found are associated with enzymatic domains and could potentially change the biochemical affinities of the proteins while not altering the active sites.

Going back to the genome annotation of CskBV (Table S2), we found that the distribution of the 17 positively selected genes was not random among the bracovirus circles (two-sided Fisher’s exact test, *p* = 0.021). Strikingly the 37.8 kb large circle 28 containing 7 of the 54 orthologs had no gene under positive selection, whereas the 7.2 kb circle 2 harbors 4 positively selected genes out 5 tested. Overall this suggests natural selection could additionally operate at the whole circle level.

### Adaptive Role of Bracovirus Genes in African *Cotesia sesamiae* Populations

Even in the particular context of bracoviruses, the detection of positive selection in homologous genes might not necessarily correlate with an effective adaptive function leading to the ecological differentiation of the parasitoid wasps. Given the challenge it would be to assess effects of particular mutations by mutational studies on parasitoid wasp, we rather investigated the molecular evolution of 3 genes in different African populations of *Cotesia sesamiae*. We chose to study the *viral Histone H4* and the *ep2* genes that among all others display the highest dN/dS ratio in the interspecies comparison and the *CrV1* gene, which has already been correlated with host range [7], [28]. These 3 genes are encoded in different bracovirus circles, namely 2, 7 and 13 respectively. We sequenced these 3 genes from 17 additional *Cotesia sesamiae* populations representing diverse ecological niches in terms of hosts and locality (Table S1).

**Figure 4:**
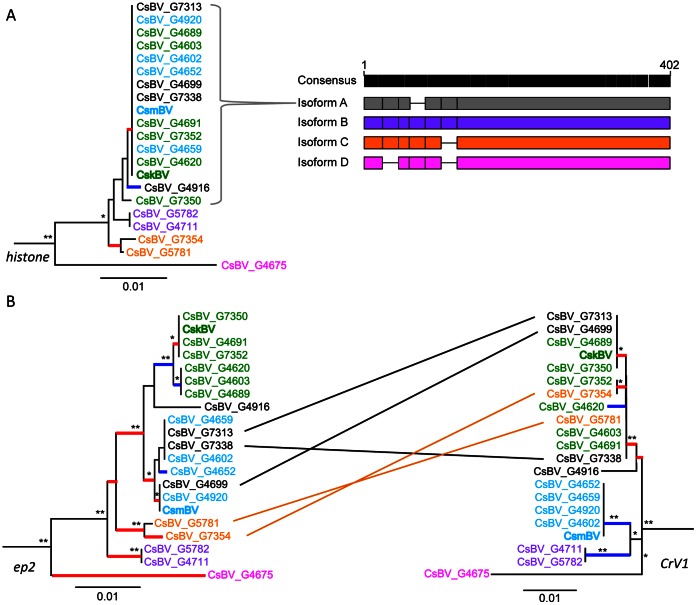
Evolution of Bracovirus *histone, ep2 and CrV1* genes in different *Cotesia sesamiae* populations. (A) Evolution of the *histone* gene: on the left, CsBV *histone* tree; on the right, genetic map showing the alignment of different isoforms, lines indicated the presence of deletions between boxed regions; (B) *ep2* (left) and *CrV1* (right) trees; lines between trees indicate the genotypes for which the 2 genes have different histories. Each genotype was assigned a color based on its main phylogenetic affiliation: purple = *histone* isoform b, orange = *histone* isoform c, pink = *histone* isoform d, green = *ep2* CskBV clade, light blue = *CrV1* CsmBV clade, and black = unassigned (refer to Table S1 for sampling details). Phylogenetic clustering is based on HKY genetic distance; ** and * indicate bootstrap support above 75 and 50 respectively; colored branches indicate results from Branch site REL analyses (HYPHY): in red with dN/dS >5 and in blue dN/dS = 0.

The *histone* tree showed that if a majority of samples were almost identical to CskBV and CsmBV, some CsBV genotypes, including G5781, G7354, G4711, G5782 and G4675, were clearly divergent (Figure 4A). The alignment revealed that the main differences corresponded to 4 isoforms showing multiple insertion and/or deletion of 7 amino acids in the first 150 nucleotides of the aligned sequences (Figure 4A). However these indels were located at least 13 AA residues upstream of DNA Binding Region, suggesting the histone could retain its function. These observations in ecologically differentiated populations could be highly significant as the N-terminal region of the *viral histone* is essential for the inhibition of host hemocyte-spreading behavior in CvBV [71]. One could hypothesize that different isoforms will have different affinities for host targets. Hence coevolution with particular host populations could select for different isoforms. Altogether this highlights the potential adaptive role of bracoviral histone for the parasitoid wasp specialization to particular host species.

The *ep2* tree is similar to the *histone* tree (Figure 4B, left). The divergent clades found with *histone* were also recovered with high bootstrap support in the *ep2* phylogeny. However, we recovered 2 additional well-supported groups corresponding to populations with similar sequences to CskBV and CsmBV respectively. In contrast, the *CrV1* phylogeny (Figure 4B, right) shows that some populations remained likewise clustered together, but that some of the genetic groups found with *histone* and *ep2* was completely lost for other populations. The clades associated with *CrV1-Coast* (CsmBV) and *CrV1-Pser* (G4711) are clearly divergent and well supported, suggesting that the 3 bracovirus loci do evolve together in these particular populations. Strikingly the populations G5781 and G7354 from Cameroon and RDC (Figure 4, in orange), harbor different haplotypes of the *CrV1-Inland* (CskBV) group, showing how poorly this marker performs on its own to distinguish these 2 populations in comparison to the other 2 genes. Moreover, 3 populations (G7313, G4699 and G7338) could not be assigned to the same groups as in the *ep2* and *histone* trees. These populations harbour CsmBV haplotypes for *ep2* and CskBV-*Inland* haplotype for *CrV1.* Since these 2 genes have been found within 37.5 kb of nucleotide distance, based on *Cotesia sesamiae kitale* BAC sequences (EF10635), this implies that recombination between neighboring loci has taken place in these populations.

When we performed branch site REL analyses to detect episodic selection [64] among the *C. sesamiae* populations, we found contrasting results between the 3 genes (Figure 4). In the *histone* tree only the 2 branches leading to the Csk/Csm clade and isoform C showed high dN/dS ratio suggesting this gene could be under positive selection in the corresponding populations (Figure 4A). In the *ep2* tree, all basal branches showed high dN/dS ratio that could be associated with diversifying selection, possibly to overcome particular host resistance. In contrast the branch leading to the CskBV clade (Figure 4B, in green) is under conservative selection (dN/dS = 0), suggesting this particular *ep2* allele is adapted to the particular ecological conditions the wasps encounter. The *CrV1* tree harbors contrasting selection patterns. The branches leading to the CsmBV clade (allele CsC) and CsBV_G4711 clade (allele CsPser) are under conservative selection, suggesting these alleles are adapted to particular host gene targets. The Csk clade however shows several branches under positive selection. These wasp populations, infesting several host species, carry different version of *CrV1* CsI alleles, and are under adaptive pressure at the *CrV1* locus. It has been shown that wasps, carrying the *CrV1* CsI allele could overcome the resistance from the host *Busseola fusca* [28]. Our results showed the high diversifying selection pressure that has occurred on the wasp populations to adapt at the *CrV1* locus, in accordance to previous findings [7]. Surprisingly, the samples G5781 and G7354 found in Central Africa (Figure 4, in orange) are evolving under high selective pressure at the 3 loci. This suggests these particular populations might be facing changes in ecological niches requiring fast adaptation at all loci.

We showed that in accordance with theoretical local adaptation framework, different wasp populations harbor different bracoviral gene haplotypes. These genes are implicated in different molecular pathway leading to host immune system disorganization. The recombination we observed between linked loci (*ep2* and *CrV1)* also suggests different haplotype combinations allow the wasp to modulate their virulence towards different lepidopteran host immune response. Altogether, this implies that natural selection on wasp populations involves several bracoviral loci.

### Conclusion

We showed molecular evolution acts differently whether we look at the viral gene arsenal of different wasp populations or different wasp species. On one hand, we found that bracovirus populations (CsBV) possessed more genes under positive selection than bracovirus species (CvBV and CcBV), showing that some bracovirus genes are involved in local host/parasite coevolution. On the other hand, the numerous multigenic families present in the PDV genomes suggest that gene duplications are also a major adaptive mechanism. We found around 60% of orthologous genes under relaxed selection pressure that could allow neo/subfunctionalization [92]. Gene duplication associated with sub-functionalization could thus play a key role in the wide viral gene arsenal of ecologically differentiated wasp populations.

This work underlines how PDVs provide a remarkable system for viral evolutionary studies, as to complete their particular life cycle they are involved in both mutualistic and pathogenic coevolutionary interactions. Here, the comparison of bracovirus genomes gave us reliable clues to understand the genetic basis of the wasp/PDV complex adaptation and specialization. We have shown diversifying selection pressures on some viral genes involved in pathogenic interactions. We identified two ways for the wasp not to get left behind in the “arms race” with its caterpillar host: (i) some of BV virulence genes were under positive selective pressure (e.g. *ep-like*, *histone H4*, *CrV1*, *lectin-C*), leading to fast modifications to optimize adaptation to host immune defenses; (ii) whereas other genes, belonging to multigenic family seem to diversify through sub-functionalization of multiple copies.

Looking within *Cotesia sesamiae* at the evolution of 3 bracovirus genes, we showed that different genes described differently the relationships of different wasp populations. This suggests that these particular genes do not carry the same selective potential for the ecologically divergent host/parasite interactions captured in the snapshot of our study. This was confirmed with the differential detection of positive selection in different wasp lineage depending on which gene was under scrutiny. Involved in multidirectional coevolutionary interactions as they parasitize many lepidopteran species, *Cotesia sesamiae* wasps rely on several molecular pathways to overcome host resistance. In the context of the geographic mosaic of coevolution [2], we can therefore conclude several bracovirus genes are responsible of the wasp local adaption. Only larger population genomic studies would reveal the intricate role PDVs play in the evolution of their carrier wasps.

## Supporting Information

Table S1 Wild *Cotesia sesamiae* populations sampled and associated gene sequence information.
(DOCX)

Table S2 Annotation of 26 circles and 7 contigs from *Cotesia sesamiae kitale* Bracovirus.
(DOCX)

Table S3 Annotation of 16 circles from *Cotesia sesamiae mombasa* Bracovirus.
(DOCX)

Table S4 Pairwise Cotesia Bracovirus Genome Comparison.
(DOCX)

Table S5 Detection of substitution saturation in the alignments of the 54 genes shared by CcBV, CskBV, CsmBV and CvBV.
(DOCX)

Table S6 Detection of substitution saturation in the pairwise alignments of the 110 genes common to CcBV and CskBV.
(DOCX)

Table S7 Annotation of the 54 orthologs shared by the 4 *Cotesia* bracoviruses.
(DOCX)

File S1 Comparative phylogenetic analyses of *Cotesia* and associated bracoviruses. Figure S1 Co-phylogeny between *Cotesia* wasps and their bracoviruses.
(DOCX)
