# Supplementary material for: Adaptive Selection on Bracovirus Genomes Drives the Specialization of Cotesia Parasitoid Wasps
Source: PLoS One. 2013 May 28;8(5):e64432. doi: 10.1371/journal.pone.0064432 (PMC3665748; doi:10.1371/journal.pone.0064432)
Supplement: Table S1 — Wild Cotesia sesamiae populations sampled and associated gene sequence information. (DOCX) [file pone.0064432.s001.docx]

Table S1: Wild *Cotesia sesamiae* populations sampled and associated gene sequence information

| **Cotesia sesamiae populations  (Gif collection)*** | **Localization** | **Lepidopteran Host Species** | **histone isoform ^a^** | **Histone accession** | **ep2 clade ^b^** | **EP2 accession** | **CrV1 allele ^c^** | **CrV1 accession** |
| --- | --- | --- | --- | --- | --- | --- | --- | --- |
| G4916 | Kenya - Endebess | *Sesamia calamistis* | *a* | JX415838 | e | JX430012 | Csend | GU549484 |
| CskBV | Kenya - Kitale | *Busseola fusca* | *a* | EF710626 | k | EF710635 | CsI | DQ356255 |
| G4689 | Kenya - Kitale 2 | *Sesamia calamistis* | *a* | JX415834 | k | JX430008 | CsI | DQ356255 |
| G7350 | Congo - Lilanda 2 | *Poeonoma sp* | *a* | JX415842 | k | JX430018 | CsI | DQ356255 |
| G4603 | Eritrea - Lamza | *Sciomesa mesophaea* | *a* | JX415829 | k | JX430003 | CsI2 | EF211978 |
| G4620 | Eritrea - Korubariya | *Pirateola piscator* | *a* | JX415830 | k | JX430004 | CsI2 | EF211978 |
| G4691 | Kenya - Kitale 2 | *Sesamia calamistis* | *a* | JX415835 | k | JX430009 | CsI2 | EF211978 |
| G7352 | Congo - Isangi 1 | *Sesamia sp nr calamistis* | *a* | JX415843 | k | JX430019 | CsI3 | KC257080 |
| G4699 | Mozambique - Nyamwerera | *Busseola nov. sp. 1* | *a* | JX415836 | m | JX430010 | CsI | DQ356255 |
| G7313 | Tanzania - Nzi | *Pirateola piscator* | *a* | KC170726 | m | JX430016 | CsI | DQ356255 |
| G7338 | Tanzania - Mwaya | *Manga sp* | *a* | JX415841 | m | JX430017 | CsI2 | EF211978 |
| CsmBV | Kenya - Mombassa | *Sesamia calamistis* | *a* | EF710636 | m | EF710642 | CsC | DQ356254 |
| G4602 | Kenya - Kisii 2 | *Sesamia nonagroides* | *a* | JX415828 | m | JX430002 | CsC | DQ356254 |
| G4652 | Kenya - Tana | *Sesamia nov. sp.5* | *a* | JX415831 | m | JX430005 | CsC | DQ356254 |
| G4659 | Kenya - Kisii 2 | *Sesamia penniseti* | *a* | JX415832 | m | JX430006 | CsC | DQ356254 |
| G4920 | South Africa - Bird Sanctuary | *Sesamia Jansei* | *a* | KC170725 | m | JX430013 | CsC | DQ356254 |
| G4711 | Cameroon - Bambuiy | *Poeonoma serrata* | *b* | JX415837 | l | JX430011 | CsPser | GU549482 |
| G5782 | Cameroon - Bamengoum | *Busseola phaia* | *b* | JX415840 | l | JX430015 | CsPser | GU549482 |
| G5781 | Cameroon - Ngombo 2 | *Poeonoma serrata* | *c* | JX415839 | r | JX430014 | CsI2 | EF211978 |
| G7354 | Republic Democratic of Congo - Pk8 Buta | *Busseola nov. sp. 2* | *c* | JX415844 | r | JX430020 | CsI3 | KC257080 |
| G4675 | Kenya - Mbita Lwanda | *Sesamia nonagroides* | *d* | JX415833 | n | JX430007 | CsSnona | GU549481 |

* Colors are the same as in figure 4

^a^ The isoforms are defined by indel position (see figure 4 A)

^b^ The ep2 clades correspond to the ep2 tree (see figure 4 B)

^c^ The CrV1 alleles were defined in Dupas (2008) and Branca (2011) and correspond to CrV1 phylogenetic clades (see figure 4 B).
