# Supplementary material for: Adaptive Selection on Bracovirus Genomes Drives the Specialization of Cotesia Parasitoid Wasps
Source: PLoS One. 2013 May 28;8(5):e64432. doi: 10.1371/journal.pone.0064432 (PMC3665748; doi:10.1371/journal.pone.0064432)
Supplement: Table S2 — Annotation of 26 circles and 7 contigs from Cotesia sesamiae kitale Bracovirus. (DOCX) [file pone.0064432.s002.docx]

Table S2: Annotation of 26 circles and 7 contigs from *Cotesia sesamiae kitale* Bracovirus

| **Circle or Contig Name** | **Circle size (bp)** | **Accession number** | **Genes †** | **Putative Function †** | **ATG position** | **STOP position** | **Sens** |
| --- | --- | --- | --- | --- | --- | --- | --- |
| **circle 1t** | 6909 partial | HF562906 | CskBV1.1 | ptp-b | 1243 | 506 | reverse |
|  |  |  | CskBV1.2 | ptp-i | 1346 | 1945 | forward |
|  |  |  | CskBV1.3 | ep1-like | 2494 | 3486 | forward |
|  |  |  | CskBV1.4 | ptp-k | 4787 | 3993 | reverse |
|  |  |  | CskBV1.5 | ptp-l | 6524 | 5649 | reverse |
| **circle 2** | 17579 | HF562907 | CskBV2.1 | ep2 | 446 | 1772 | forward |
|  |  |  | CskBV2.2 | hypothetical protein | 3449 | 4411 | forward |
|  |  |  | CskBV2.3 | hypothetical protein | 5286 | 6125 | forward |
|  |  |  | CskBV2.4 | bv15-like | 7508 | 8115 | forward |
|  |  |  | CskBV2.5 | bv2-like | 10673 | 11999 | forward |
|  |  |  | CskBV2.6 | bv14-like | 13107 | 13943 | forward |
|  |  |  | CskBV2.7 | conserved hypothetical protein | 15779 | 16833 | forward |
| **circle 4t** | 7333 partial | HF562908 | CskBV4.1 | bv12-like | 1606 | 454 | reverse |
|  |  |  | CskBV4.2 | ptp-h | 3737 | 2775 | reverse |
|  |  |  | CskBV4.3 | ptp-o | 4889 | 5794 | forward |
| **circle 5t** | 10204 partial | HF562909 | CskBV5.2 | bv16-like | 4378 | 3870 | reverse |
|  |  |  | CskBV5.3 | bv16-like | 6287 | 5788 | reverse |
|  |  |  | CskBV5.4 | ep1-like | 9264 | 8210 | reverse |
| **circle 6** | 10843 | HF562910 | CskBV6.1 | ben domain protein | 2067 | 4270 | forward |
|  |  |  | CskBV6.2 | conserved hypothetical protein | 7691 | 8270 | forward |
|  |  |  | CskBV6.3 | conserved hypothetical protein | 9508 | 9948 | forward |
| **circle 7** | 18861 | HF562911 | CskBV7.1 | histone H4-like | 586 | 969 | forward |
|  |  |  | CskBV7.2 | ptp-r | 2848 | 1883 | reverse |
|  |  |  | CskBV7.3 | ep1-like | 5143 | 3906 | reverse |
|  |  |  | CskBV7.4 | p94 | 5989 | 8463 | forward |
|  |  |  | CskBV7.5 | p94-like | 10157 | 10801 | forward |
|  |  |  | CskBV7.6 | ep1-like | 11670 | 11122 | reverse |
|  |  |  | CskBV7.7 | p94-like | 12662 | 13726 | forward |
| **circle 8/21t** | 5362 | HF562912 | CskBV8.1 | ep1-like | 3699 | 2206 | reverse |
| **circle 12t** | 4356 | HF562913 | CskBV12.1 | bv8-like | 1953 | 2691 | forward |
|  |  |  | CskBV12.2 | conserved hypothetical protein | 3600 | 3842 | forward |
| **circle 13** | 21050 | HF562914 | CskBV13.1 | bv19-like | 259 | 987 | forward |
|  |  |  | CskBV13.2 | bv3-like | 1673 | 2489 | forward |
|  |  |  | CskBV13.3 | conserved hypothetical protein | 3559 | 4401 | forward |
|  |  |  | CskBV13.4 | bv14-like | 4788 | 5879 | forward |
|  |  |  | CskBV13.5 | crv1 | 9687 | 10898 | forward |
|  |  |  | CskBV13.6 | conserved hypothetical protein | 13502 | 14105 | forward |
|  |  |  | CskBV13.7 | conserved hypothetical protein | 14906 | 15268 | forward |
|  |  |  | CskBV13.8 | bv19-like | 16411 | 18511 | forward |
|  |  |  | CskBV13.9 | lectin c-type | 20442 | 19823 | reverse |
| **circle 14t** | 9189 partial | HF562915 | CskBV14.1 | viral ankyrin ank3 | 1276 | 770 | reverse |
|  |  |  | CskBV14.2 | conserved hypothetical protein | 2758 | 3391 | forward |
|  |  |  | CskBV14.3 | ptp-v | 4697 | 5611 | forward |
|  |  |  | CskBV14.4 | ptp-omega | 6000 | 6917 | forward |
|  |  |  | CskBV14.5 | ptp-u | 8332 | 9162 | forward |
| **Circle 15** | 732 | HF562916 | No ORFs |  |  |  |  |
| **circle 16t** | 7265 partial | HF562917 | CskBV16.1 | viral ankyrin ank7 | 2478 | 1948 | reverse |
|  |  |  | CskBV16.2 | viral ankyrin ank8 | 3717 | 3202 | reverse |
|  |  |  | CskBV16.3 | bv8 | 4902 | 5370 | forward |
|  |  |  | CskBV16.4 | bv6 | 7040 | 6720 | reverse |
| **circle 17** | 14577 | HF562918 | CskBV17.1 | ptp-alpha | 1618 | 2529 | forward |
|  |  |  | CskBV17.2 | bv12-like | 4368 | 3209 | reverse |
|  |  |  | CskBV17.3 | ptp-Z | 5467 | 6303 | forward |
|  |  |  | CskBV17.4 | ptp-n | 8371 | 9321 | forward |
|  |  |  | CskBV17.5 | ptp-e/x | 10247 | 11212 | forward |
|  |  |  | CskBV17.6 | ptp-tau | 13073 | 13906 | forward |
| **circle 18t** | 10425 partial | HF562919 | CskBV18.1 | conserved hypothetical protein | 580 | 1553 | forward |
|  |  |  | CskBV18.2 | bv6-26-like | 6742 | 6470 | reverse |
|  |  |  | CskBV18.3 | ser-rich6-like protein | 7196 | 7564 | forward |
|  |  |  | CskBV18.4 | bv6-25-like protein | 9801 | 9517 | reverse |
| **circle 23** | 13070 | HF562920 | CskBV23.1 | unknown hypothetical protein | 989 | 1537 | forward |
|  |  |  | CskBV23.2 | bv5 | 5242 | 4715 | reverse |
|  |  |  | CskBV23.3 | RNase T2-like | 5715 | 7443 | forward |
|  |  |  | CskBV23.4 | unknown hypothetical protein | 8701 | 8236 | reverse |
|  |  |  | CskBV23.5 | ben domain protein | 9153 | 12477 | forward |
| **circle 24** | 21120 | HF562921 | CskBV24.1 | bv18 | 1204 | 1676 | forward |
|  |  |  | CskBV24.2 | conserved hypothetical protein | 6543 | 6217 | reverse |
|  |  |  | CskBV24.3 | ben domain protein | 9940 | 12624 | forward |
|  |  |  | CskBV24.4 | conserved hypothetical protein | 15080 | 16226 | forward |
|  |  |  | CskBV24.5 | ben domain protein | 17209 | 19952 | forward |
| **circle 25t** | 10493 partial | HF562921 | CskBV25.1 | bv2 | 495 | <2 | reverse |
|  |  |  | CskBV25.2 | RNase T2-like | 1430 | 2594 | forward |
|  |  |  | CskBV25.3 | RNase T2-like | 4154 | 5884 | forward |
|  |  |  | CskBV25.4 | ben domain protein | 7207 | 9666 | forward |
| **circle 26t** | 10544 partial | HF562923 | CskBV26.1 | ptp-a | 424 | 1401 | forward |
|  |  |  | CskBV26.2 | ptp-epsilon | 2689 | 1684 | reverse |
|  |  |  | CskBV26.3 | ptp-delta | 4536 | 3568 | reverse |
|  |  |  | CskBV26.4 | ptp-kappa | 6460 | 7440 | forward |
|  |  |  | CskBV26.5 | viral ankyrin ank6 | 8192 | 8680 | forward |
| **circle 27** | 26866 | HF562924 | CskBV27.1 | bv8 | 8369 | 8961 | forward |
|  |  |  | CskBV27.2 | ben domain protein | 17245 | 20990 | forward |
|  |  |  | CskBV27.3 | ben domain protein | 23013 | 25577 | forward |
| **circle 28** | 37873 | HF562925 | CskBV28.1 | conserved hypothetical protein | 760 | 1253 | forward |
|  |  |  | CskBV28.2 | conserved hypothetical protein | 2857 | 3141 | forward |
|  |  |  | CskBV28.3 | conserved hypothetical protein | 4341 | 6750 | forward |
|  |  |  | CskBV28.4 | conserved hypothetical protein | 8098 | 7312 | reverse |
|  |  |  | CskBV28.5 | bv9 | 9484 | 10225 | forward |
|  |  |  | CskBV28.6 | bv10 | 10746 | 11036 | forward |
|  |  |  | CskBV28.7 | bv6 | 12885 | 12616 | reverse |
|  |  |  | CskBV28.8 | ser-rich protein | 13255 | 13707 | forward |
|  |  |  | CskBV28.9 | bv23 | 14201 | 14443 | forward |
|  |  |  | CskBV28.10 | bv6 | 16579 | 16307 | reverse |
|  |  |  | CskBV28.11 | bv8 | 17215 | 17699 | forward |
|  |  |  | CskBV28.12 | bv9 | 19702 | 20195 | forward |
|  |  |  | CskBV28.13 | ser-rich protein | 21017 | 23339 | forward |
|  |  |  | CskBV28.14 | bv8 | 28096 | 28627 | forward |
|  |  |  | CskBV28.15 | conserved hypothetical protein | 30639 | 31004 | forward |
|  |  |  | CskBV28.16 | bv6 | 31647 | 31345 | reverse |
|  |  |  | CskBV28.17 | bv6 | 37121 | 36780 | reverse |
| **circle 30t** | 10893 partial | HF562926 | CskBV30.1 | bv11 | 312 | 1868 | forward |
|  |  |  | CskBV30.2 | bv3 | 4220 | 4997 | forward |
|  |  |  | CskBV30.3 | bv14 | 7821 | 8456 | forward |
| **circle 32** | 34148 | HF562927 | CskBV32.1 | bv21 | 1096 | 803 | reverse |
|  |  |  | CskBV32.2 | bv6 | 2297 | 1983 | reverse |
|  |  |  | CskBV32.3 | bv9 | 2998 | 4011 | forward |
|  |  |  | CskBV32.4 | bv10 | 5251 | 5541 | forward |
|  |  |  | CskBV32.5 | bv6 | 6411 | 6046 | reverse |
|  |  |  | CskBV32.6 | hypothetical protein | 9624 | 10156 | forward |
|  |  |  | CskBV32.7 | bv8 | 11267 | 12242 | forward |
|  |  |  | CskBV32.8 | hypothetical protein | 13343 | 13603 | forward |
|  |  |  | CskBV32.9 | bv6 | 15308 | 15039 | reverse |
|  |  |  | CskBV32.10 | bv6 | 16413 | 16141 | reverse |
|  |  |  | CskBV32.11 | conserved hypothetical protein | 17517 | 18375 | forward |
|  |  |  | CskBV32.12 | conserved hypothetical protein | 22897 | 23652 | forward |
|  |  |  | CskBV32.13 | crp3 | 28335 | 28774 | forward |
|  |  |  | CskBV32.14 | bv6 | 30749 | 30282 | reverse |
|  |  |  | CskBV32.15 | ser-rich protein | 31782 | 32063 | forward |
|  |  |  | CskBV32.16 | bv6 | 33530 | 33258 | reverse |
| **circle 33** | 24455 | HF562928 | CskBV33.1 | bv5 | 1431 | 794 | reverse |
|  |  |  | CskBV33.2 | bv5 | 4008 | 3523 | reverse |
|  |  |  | CskBV33.3 | bv1 | 8339 | 8950 | forward |
|  |  |  | CskBV33.4 | ben domain protein | 10399 | 12561 | forward |
|  |  |  | CskBV33.5 | ben domain protein | 15747 | 19261 | forward |
| **circle 35** | 14172 | HF562929 | CskBV35.1 | bv8 | 2365 | 3474 | forward |
|  |  |  | CskBV35.2 | bv6 | 4261 | 4013 | reverse |
|  |  |  | CskBV35.3 | crp | 7288 | 8015 | forward |
| **circle 36** | 11409 | HF562930 | CskBV36.1 | bv7 | 1503 | 748 | reverse |
|  |  |  | CskBV36.2 | hypothetical protein | 1854 | 3047 | forward |
|  |  |  | CskBV36.3 | Putative capsid-like protein | 4952 | 4364 | reverse |
|  |  |  | CskBV36.4 | bv7 | 6857 | 6096 | reverse |
|  |  |  | CskBV36.5 | bv11 | 9770 | 8499 | reverse |
| **circle 37** | 3307 | HF562931 | CskBV37.1 | ep1-like | 2046 | 1306 | reverse |
| Contig c159 | 668 | HS570923 | CskBV_c159.1 | ptp-q | 3 | 617 | forward |
| Contig c46 | 1306 | HS570924 | CskBV_c46.1 | ptp7-like | 1305 | 538 | reverse |
| Contig c56 | 1205 | HS570925 | CskBV_c56.1 | ptp-theta | 1203 | 334 | reverse |
| Contig c5 | 4361 | HS570926 | CskBV_c5.1 | ptp-zeta | 132 | 1022 | forward |
|  |  |  | CskBV_c5.2 | ptp-y | 3095 | 2184 | reverse |
| Contig c99 | 863 | HS570927 | CskBV_c99.1 | viral ankyrin ank1-like | 85 | 552 | forward |
| Contig cg1.1 | 771 | HS570928 | CskBV_cg1.1 | cystatin-a | 198 | 617 | forward |
| Contig cg2.1 | 741 | HS570929 | CskBV_cg2.1 | cystatin-b | 408 | 1 | reverse |

† Colored genes represent the 54 orthologous dataset used for detecting positive selection: positively selected in red, negatively or neutrally selected in blue.
