# Supplementary material for: Adaptive Selection on Bracovirus Genomes Drives the Specialization of Cotesia Parasitoid Wasps
Source: PLoS One. 2013 May 28;8(5):e64432. doi: 10.1371/journal.pone.0064432 (PMC3665748; doi:10.1371/journal.pone.0064432)
Supplement: Table S3 — Annotation of 16 circles from Cotesia sesamiae mombasa Bracovirus. (DOCX) [file pone.0064432.s003.docx]

Table S3: Annotation of 16 circles from *Cotesia sesamiae mombasa* Bracovirus

| **Segment Name** | **Circle size (bp)** | **Accession number** | **Genes †** | **Putative Function †** | **BAC** | **ATG position** | **STOP position** | **Sens** |
| --- | --- | --- | --- | --- | --- | --- | --- | --- |
| **circle 2** | 16670 | EF710642 | CsmBV2.1 | ep2 | 14g12 | 100473 | 99146 | reverse |
|  |  |  | CsmBV2.2 | hypothetical protein |  | 97475 | 96513 | reverse |
|  |  |  | CsmBV2.3 | hypothetical protein |  | 95636 | 94797 | reverse |
|  |  |  | CsmBV2.4 | bv15-like |  | 93408 | 92796 | reverse |
|  |  |  | CsmBV2.5 | bv2-like |  | 90245 | 88919 | reverse |
|  |  |  | CsmBV2.6 | bv14-like |  | 87574 | 86747 | reverse |
|  |  |  | CsmBV2.7 | hypothetical protein |  | 86055 | 85001 | reverse |
| **circle 7** | 18861 | EF710636 | CsmBV7.1 | histone H4-like | 1c22 | 86783 | 86400 | reverse |
|  |  |  | CsmBV7.2 | ptp-r |  | 84507 | 85472 | forward |
|  |  |  | CsmBV7.3 | ep1-like |  | 82876 | 83445 | forward |
|  |  |  | CsmBV7.4 | p94 |  | 81360 | 78886 | reverse |
|  |  |  | CsmBV7.5 | hypothetical protein |  | 77188 | 76544 | reverse |
|  |  |  | CsmBV7.6 | ep1-like |  | 75675 | 76223 | forward |
|  |  |  | CsmBV7.7 | p94-like |  | 74683 | 73619 | reverse |
| **circle 13** | 25094 | EF710642 | CsmBV13.1 | ep2-like1 | 14g12 | 67196 | 66468 | reverse |
|  |  |  | CsmBV13.2 | bv3-like |  | 64751 | 63930 | reverse |
|  |  |  | CsmBV13.10 | hypothetical protein |  | 63040 | 61271 | reverse |
|  |  |  | CsmBV13.3 | hypothetical protein |  | 60058 | 59216 | reverse |
|  |  |  | CsmBV13.4 | bv14-like |  | 58829 | 57570 | reverse |
|  |  |  | CsmBV13.5 | crv1 |  | 53786 | 52584 | reverse |
|  |  |  | CsmBV13.6 | hypothetical protein |  | 49941 | 49322 | reverse |
|  |  |  | CsmBV13.7 | hypothetical protein |  | 48521 | 47799 | reverse |
|  |  |  | CsmBV13.8 | bv19-like |  | 47008 | 44908 | reverse |
|  |  |  | CsmBV13.9 | lectin c-type |  | 42974 | 43588 | forward |
| **Circle 15** | 691 | EF710638 | No ORFs |  |  |  |  |  |
| **circle 16t** | 7706 partial | EF710641 | CsmBV16.1 | viral ankyrin ank7 | 6l7 | 5230 | 5760 | forward |
|  |  |  | CsmBV16.2 | viral ankyrin ank8 |  | 3991 | 4506 | forward |
|  |  |  | CsmBV16.3 | bv8 |  | 2061 | 1593 | reverse |
|  |  |  | CsmBV16.5 | hypothetical protein |  | 499 | 2 | reverse |
| **circle 17** | 19784 | EF710639 | CsmBV17.1 | ptp-alpha | 4b19 | 44232 | 45143 | forward |
|  |  |  | CsmBV17.2 | bv12-like |  | 46976 | 45817 | reverse |
|  |  |  | CsmBV17.3 | ptp-Z |  | 48075 | 48986 | forward |
|  |  |  | CsmBV17.4 | ptp-n |  | 49822 | 50772 | forward |
|  |  |  | CsmBV17.5 | ptp-x |  | 51734 | 52677 | forward |
|  |  |  | CsmBV17.7 | ptp-h |  | 59997 | 60677 | forward |
|  |  |  | CsmBV17.6 | ptp-tau |  | 61356 | 62189 | forward |
| **circle 18** | 24396 | EF710641 | CsmBV18.1 | hypothetical protein | 6l7 | 9051 | 10024 | forward |
|  |  |  | CsmBV18.6 | BEN domain protein |  | 14008 | 15603 | forward |
|  |  |  | CsmBV18.5 | hypothetical BEN domain protein |  | 16870 | 18185 | forward |
|  |  |  | CsmBV18.2 | bv6-26-like |  | 28082 | 27810 | reverse |
|  |  |  | CsmBV18.3 | ser-rich6-like protein |  | 28557 | 28904 | forward |
|  |  |  | CsmBV18.4 | bv6-25-like protein |  | 32224 | 31940 | reverse |
| **circle 24** | 21104 | EF710641 | CsmBV24.1 | bv18 | 6l7 | 55461 | 55933 | forward |
|  |  |  | CsmBV24.2 | hypothetical protein |  | 60798 | 60472 | reverse |
|  |  |  | CsmBV24.3 | ben domain protein |  | 64197 | 66877 | forward |
|  |  |  | CsmBV24.4 | hypothetical protein |  | 69335 | 70495 | forward |
|  |  |  | CsmBV24.5 | ben domain protein |  | 71478 | 74194 | forward |
| **circle 26t** | 13439 partial | EF710640 | CsmBV26.1 | ptp-a | 6i18 | 12979 | 12002 | reverse |
|  |  |  | CsmBV26.2 | ptp-epsilon |  | 10726 | 11730 | forward |
|  |  |  | CsmBV26.3 | ptp-delta |  | 8727 | 9686 | forward |
|  |  |  | CsmBV26.4 | ptp-kappa |  | 6746 | 5766 | reverse |
|  |  |  | CsmBV26.5 | viral ankyrin ank6 |  | 4479 | 3991 | reverse |
| **circle 27** | 26887 | EF710638 | CsmBV27.1 | bv8 | 2a4 | 74754 | 75346 | forward |
|  |  |  | CsmBV27.2 | ben domain protein |  | 83644 | 87383 | forward |
|  |  |  | CsmBV27.3 | ben domain protein |  | 89415 | 91979 | forward |
| **circle 28** | 37669 | EF710642 | CsmBV28.1 | hypothetical protein | 14g12 | 899 | 1395 | forward |
|  |  |  | CsmBV28.2 | hypothetical protein |  | 3272 | 2988 | reverse |
|  |  |  | CsmBV28.3 | hypothetical protein |  | 4499 | 6909 | forward |
|  |  |  | CsmBV28.4 | hypothetical protein |  | 8258 | 7472 | reverse |
|  |  |  | CsmBV28.5 | bv9 |  | 9422 | 10163 | forward |
|  |  |  | CsmBV28.6 | bv10 |  | 10685 | 10975 | forward |
|  |  |  | CsmBV28.7 | bv6 |  | 12821 | 12552 | reverse |
|  |  |  | CsmBV28.8 | ser-rich protein |  | 13191 | 13643 | forward |
|  |  |  | CsmBV28.9 | bv23 |  | 14137 | 14379 | forward |
|  |  |  | CsmBV28.10 | bv6 |  | 16510 | 16238 | reverse |
|  |  |  | CsmBV28.11 | bv8 |  | 17146 | 17629 | forward |
|  |  |  | CsmBV28.12 | bv9 |  | 19631 | 20127 | forward |
|  |  |  | CsmBV28.13 | ser-rich protein |  | 20949 | 23271 | forward |
|  |  |  | CsmBV28.14 | bv8 |  | 28027 | 28558 | forward |
|  |  |  | CsmBV28.15 | hypothetical protein |  | 30574 | 30939 | forward |
|  |  |  | CsmBV28.16 | bv6 |  | 31581 | 31279 | reverse |
|  |  |  | CsmBV28.17 | bv6 |  | 37050 | 36709 | reverse |
| **circle 32** | 32737 | EF710638 | CsmBV32.1 | bv21 | 2a4 | 33711 | 33418 | reverse |
|  |  |  | CsmBV32.2 | bv6 |  | 34912 | 34598 | reverse |
|  |  |  | CsmBV32.3 | bv9 |  | 35613 | 36626 | forward |
|  |  |  | CsmBV32.4 | bv10 |  | 37867 | 38154 | forward |
|  |  |  | CsmBV32.5 | bv6 |  | 39024 | 38659 | reverse |
|  |  |  | CsmBV32.6 | hypothetical protein |  | 42246 | 42778 | forward |
|  |  |  | CsmBV32.7 | bv8 |  | 43889 | 44875 | forward |
|  |  |  | CsmBV32.8 | hypothetical protein |  | 45975 | 46235 | forward |
|  |  |  | CsmBV32.9 | bv6 |  | 47921 | 47652 | reverse |
|  |  |  | CsmBV32.10 | bv6 |  | 49026 | 48754 | reverse |
|  |  |  | CsmBV32.11 | hypothetical protein |  | 50129 | 50987 | forward |
|  |  |  | CsmBV32.12 | hypothetical protein |  | 54340 | 55095 | forward |
|  |  |  | CsmBV32.13 | crp3 |  | 59545 | 59985 | forward |
|  |  |  | CsmBV32.14 | bv6 |  | 61960 | 61493 | reverse |
|  |  |  | CsmBV32.15 | ser-rich protein |  | 62993 | 63361 | forward |
|  |  |  | CsmBV32.16 | bv6 |  | 64734 | 64450 | reverse |
| **circle 33t** | 3001 partial | EF710642 | CsmBV33.1 | bv5 | 14g12 | 102468 | 101831 | reverse |
| **circle 35** | 21127 | EF710641 | CsmBV35.1 | bv8 | 6l7 | 39970 | 39243 | reverse |
|  |  |  | CsmBV35.2 | bv6 |  | 50123 | 50371 | forward |
|  |  |  | CsmBV35.3 | crp |  | 52019 | 50910 | reverse |
| **circle 36** | 16467 | EF710642 | CsmBV36.1 | bv7 | 14g12 | 69180 | 68425 | reverse |
|  |  |  | CsmBV36.2 | hypothetical protein |  | 69531 | 70721 | forward |
|  |  |  | CsmBV36.3 | Putative capsid-like protein |  | 75739 | 75150 | reverse |
|  |  |  | CsmBV36.4 | bv7 |  | 77644 | 76883 | reverse |
|  |  |  | CsmBV36.5 | bv11 |  | 82509 | 81238 | reverse |
| **circle 37** | 3624 | EF710642 | CsmBV37.1 | ep1-like | 14g12 | 39634 | 40374 | forward |
