# Supplementary material for: Adaptive Selection on Bracovirus Genomes Drives the Specialization of Cotesia Parasitoid Wasps
Source: PLoS One. 2013 May 28;8(5):e64432. doi: 10.1371/journal.pone.0064432 (PMC3665748; doi:10.1371/journal.pone.0064432)
Supplement: Table S4 — Pairwise Cotesia Bracovirus Genome Comparison. (DOCX) [file pone.0064432.s004.docx]

Table S4: Pairwise Cotesia Bracovirus Genome Comparison

| **BV pair** | **# orthologs** | **% orthologs** | **% AA identity** | **% positive** |
| --- | --- | --- | --- | --- |
| CcBV / CvBV | 108 | 48 / 68 | 77.4 | 86.8 |
| CcBV / CskBV | 105 | 47 / 76 | 75 | 85.3 |
| CcBV / CsmBV | 65 | 29 / 74 | 75.6 | 85.5 |
| CskBV / CsmBV | 79 | 57 / 90 | 96.8 | 98.2 |
| CskBV / CvBV | 102 | 73 / 65 | 83.3 | 90.4 |
| CsmBV / CvBV | 74 | 85 / 47 | 82.4 | 90.1 |
