# Supplementary material for: Adaptive Selection on Bracovirus Genomes Drives the Specialization of Cotesia Parasitoid Wasps
Source: PLoS One. 2013 May 28;8(5):e64432. doi: 10.1371/journal.pone.0064432 (PMC3665748; doi:10.1371/journal.pone.0064432)
Supplement: Table S5 — Detection of substitution saturation in the alignments of the 54 genes shared by CcBV, CskBV, CsmBV and CvBV. (DOCX) [file pone.0064432.s005.docx]

Table S5: Detection of substitution saturation in the alignments of the 54 genes shared by CcBV, CskBV, CsmBV and CvBV

| **Gene (CcBV nomenclature)** | **Observed saturation Index (Iss)** | **Expected saturation index (Iss.c -Sym)** | **T** | **DF** | ***p*-value (2-tailed) ^a^** |
| --- | --- | --- | --- | --- | --- |
| CcBV_18.2 | 0,124 | 0,809 | 53,94 | 740 | < 10^-4^ |
| CcBV_13.2 | 0,241 | 0,792 | 23,79 | 362 | < 10^-4^ |
| ank-6_(CcBV_26.3b) | 0,090 | 0,803 | 56,53 | 509 | < 10^-4^ |
| ank-7_(CcBV_16.1) | 0,109 | 0,787 | 41,20 | 386 | < 10^-4^ |
| ank-8_(CcBV_16.2) | 0,077 | 0,787 | 44,85 | 308 | < 10^-4^ |
| ben-2_(CcBV_3.4) | 0,116 | 0,849 | 126,76 | 3440 | < 10^-4^ |
| ben-14_(CcBV_24.1) | 0,151 | 0,839 | 79,70 | 1895 | < 10^-4^ |
| bv11-4_(CcBV_36.3) | 0,202 | 0,817 | 44,07 | 920 | < 10^-4^ |
| bv12-2_(CcBV_10.5b) | 0,168 | 0,798 | 37,42 | 530 | < 10^-4^ |
| bv14-2_(CcBV_18.3) | 0,135 | 0,823 | 63,24 | 1076 | < 10^-4^ |
| bv15-2_(CcBV_2.5) | 0,148 | 0,792 | 35,58 | 452 | < 10^-4^ |
| bv18-2_(CcBV_27.6) | 0,189 | 0,777 | 20,22 | 197 | < 10^-4^ |
| bv19.2_(CcBV_13.1b) | 0,109 | 0,796 | 47,48 | 512 | < 10^-4^ |
| bv2-7_(CcBV_31.4) | 0,176 | 0,805 | 39,32 | 659 | < 10^-4^ |
| bv21-2_(CcBV_32.12) | 0,145 | 0,786 | 30,46 | 290 | < 10^-4^ |
| bv5-3_(CcBV_9.5) | 0,249 | 0,789 | 23,66 | 410 | < 10^-4^ |
| bv6-5_(CcBV_29.13) | 0,072 | 0,779 | 40,66 | 269 | < 10^-4^ |
| bv6-17_(CcBV_32.14) | 0,193 | 0,783 | 23,93 | 329 | < 10^-4^ |
| bv6-19_(CcBV_32.16) | 0,091 | 0,778 | 35,85 | 260 | < 10^-4^ |
| bv6-24_(CcBV_35.1a) | 0,138 | 0,777 | 26,73 | 245 | < 10^-4^ |
| bv6-25_(CcBV_18.9b) | 0,102 | 0,779 | 35,40 | 281 | < 10^-4^ |
| bv6-26_(CcBV_18.11) | 0,072 | 0,779 | 42,21 | 269 | < 10^-4^ |
| bv8-3_(CcBV_28.6) | 0,323 | 0,783 | 17,54 | 326 | < 10^-4^ |
| bv8-6like_(CcBV15.4blike) | 0,156 | 0,789 | 32,69 | 407 | < 10^-4^ |
| bv8-11_(CcBV_16.3) | 0,105 | 0,780 | 32,98 | 230 | < 10^-4^ |
| bv9-5_(CcBV_28.10) | 0,159 | 0,785 | 30,19 | 356 | < 10^-4^ |
| bv9-6_(CcBV_28.18) | 0,135 | 0,779 | 31,15 | 281 | < 10^-4^ |
| CcBV_18.13 | 0,163 | 0,790 | 33,06 | 422 | < 10^-4^ |
| CcBV_24.2 | 0,235 | 0,807 | 34,28 | 701 | < 10^-4^ |
| CcBV_32.6 | 0,123 | 0,810 | 54,36 | 752 | < 10^-4^ |
| CcBV_32.7b | 0,150 | 0,779 | 27,93 | 281 | < 10^-4^ |
| crp3_(CcBV_32.3) | 0,172 | 0,781 | 27,43 | 308 | < 10^-4^ |
| CcV1_(CcBV_13.3) | 0,194 | 0,823 | 50,13 | 1067 | < 10^-4^ |
| CcBV_31.11 | 0,222 | 0,808 | 35,60 | 710 | < 10^-4^ |
| bv11-3_(CcBV_2.2) | 0,174 | 0,819 | 50,65 | 962 | < 10^-4^ |
| bv9-7_(CcBV_28.21) | 0,096 | 0,789 | 44,27 | 404 | < 10^-4^ |
| bv6-8_(CcBV_29.21) | 0,096 | 0,779 | 36,02 | 269 | < 10^-4^ |
| ep1-like4_(CcBV_5.5) | 0,386 | 0,793 | 17,15 | 464 | < 10^-4^ |
| ep1-like6_(CcBV_28.1) | 0,201 | 0,809 | 40,50 | 734 | < 10^-4^ |
| ep2_(CcBV_2.4) | 0,222 | 0,815 | 40,70 | 851 | < 10^-4^ |
| histone_(CcBV_7.3) | 0,155 | 0,794 | 32,89 | 380 | < 10^-4^ |
| CcV3_(CcBV_13.5) | 0,120 | 0,794 | 41,26 | 470 | < 10^-4^ |
| p94-like1_(CcBV_7.1b) | 0,255 | 0,832 | 49,99 | 1382 | < 10^-4^ |
| p94-like2_(CcBV_7.2b) | 0,106 | 0,822 | 71,75 | 1043 | < 10^-4^ |
| ptpa_(CcBV_26.6) | 0,096 | 0,819 | 71,64 | 965 | < 10^-4^ |
| ptpalpha_(CcBV_17.4) | 0,096 | 0,819 | 71,64 | 965 | < 10^-4^ |
| ptpdelta_(CcBV_26.1) | 0,110 | 0,819 | 66,73 | 956 | < 10^-4^ |
| ptpe_(CcBV_10.1) | 0,292 | 0,800 | 25,41 | 578 | < 10^-4^ |
| ptph_(CcBV_4.2) | 0,292 | 0,800 | 25,41 | 578 | < 10^-4^ |
| ptpr_(CcBV_7.4) | 0,082 | 0,819 | 77,95 | 956 | < 10^-4^ |
| ptpt_(CcBV_10.3) | 0,110 | 0,785 | 38,98 | 359 | < 10^-4^ |
| ptpz_(CcBV_17.3) | 0,088 | 0,813 | 67,47 | 815 | < 10^-4^ |
| ser-rich5_(CcBV_28.14) | 0,105 | 0,792 | 44,01 | 446 | < 10^-4^ |
| ser-rich8_(CcBV_18.10) | 0,094 | 0,777 | 33,14 | 242 | < 10^-4^ |

^a^ Statistical significance of the difference between observed and expected saturation indexes
