# Supplementary material for: Adaptive Selection on Bracovirus Genomes Drives the Specialization of Cotesia Parasitoid Wasps
Source: PLoS One. 2013 May 28;8(5):e64432. doi: 10.1371/journal.pone.0064432 (PMC3665748; doi:10.1371/journal.pone.0064432)
Supplement: Table S6 — Detection of substitution saturation in the pairwise alignments of the 110 genes common to CcBV and CskBV. (DOCX) [file pone.0064432.s006.docx]

Table S6: Detection of substitution saturation in the pairwise alignments of the 110 genes common to CcBV and CskBV.

| **Gene (CcBV nomenclature)** | **Observed saturation Index (Iss)** | **Expected saturation index (Iss.c -Sym)** | **T** | **DF** | ***p*-value (2-tailed) ^a^** |
| --- | --- | --- | --- | --- | --- |
| ccbv_18.2 | 0,175 | 0,830 | 53,064 | 740 | < 10^-4^ |
| ccbv_13.2 | 0,293 | 0,808 | 23,876 | 362 | < 10^-4^ |
| ccbv_22.4c | 0,414 | 0,836 | 28,317 | 959 | < 10^-4^ |
| ank-1__ccbv_11.1_ | 0,126 | 0,817 | 51,379 | 464 | < 10^-4^ |
| ank-3__ccbv_14.2_ | 0,144 | 0,820 | 48,972 | 503 | < 10^-4^ |
| ank-6__ccbv_26.3b_ | 0,292 | 0,818 | 28,057 | 485 | < 10^-4^ |
| ank-7__ccbv_16.1_ | 0,086 | 0,803 | 51,593 | 311 | < 10^-4^ |
| ank-8__ccbv_16.2_ | 0,115 | 0,820 | 56,990 | 512 | < 10^-4^ |
| ben-10__ccbv_25.2b_ | 0,202 | 0,845 | 85,822 | 2249 | < 10^-4^ |
| ben-2__ccbv_3.4_ | 0,183 | 0,845 | 116,203 | 3581 | < 10^-4^ |
| ben-14__ccbv_24.1_ | 0,255 | 0,846 | 73,576 | 2369 | < 10^-4^ |
| ben-11__ccbv_33.2_ | 0,216 | 0,843 | 70,803 | 1700 | < 10^-4^ |
| ben-8__ccbv_20.2_ | 0,208 | 0,846 | 101,504 | 3272 | < 10^-4^ |
| bv1-1__ccbv_9.2_ | 0,287 | 0,817 | 27,840 | 461 | < 10^-4^ |
| bv10-2__ccbv_28.17_ | 0,220 | 0,800 | 26,556 | 287 | < 10^-4^ |
| bv10-3__ccbv_32.24_ | 0,261 | 0,793 | 20,932 | 242 | < 10^-4^ |
| bv11-1__ccbv_30.6b_ | 0,496 | 0,843 | 28,641 | 1586 | < 10^-4^ |
| bv11-4__ccbv_36.3_ | 0,371 | 0,837 | 33,611 | 1016 | < 10^-4^ |
| bv12-2__ccbv_10.5b_ | 0,274 | 0,826 | 34,489 | 629 | < 10^-4^ |
| bv12-3__ccbv_4.1a_ | 0,235 | 0,826 | 39,330 | 629 | < 10^-4^ |
| bv14-2__ccbv_18.3_ | 0,215 | 0,839 | 56,566 | 1094 | < 10^-4^ |
| bv14-1__ccbv_30.2_ | 0,167 | 0,826 | 50,362 | 632 | < 10^-4^ |
| bv15-2__ccbv_2.5_ | 0,215 | 0,816 | 34,979 | 452 | < 10^-4^ |
| bv16-1__ccbv_5.2_ | 0,209 | 0,812 | 33,176 | 398 | < 10^-4^ |
| bv16-2__ccbv_5.3_ | 0,204 | 0,810 | 32,874 | 380 | < 10^-4^ |
| bv18-2__ccbv_27.6_ | 0,221 | 0,784 | 21,476 | 197 | < 10^-4^ |
| ep2-like5__ccbv_13like.4b_ | 0,327 | 0,820 | 26,212 | 518 | < 10^-4^ |
| bv19.2__ccbv_13.1b_ | 0,223 | 0,823 | 38,964 | 578 | < 10^-4^ |
| bv2-7__ccbv_31.4_ | 0,388 | 0,835 | 30,154 | 929 | < 10^-4^ |
| bv2-5__ccbv_25.3_ | 0,302 | 0,811 | 24,395 | 392 | < 10^-4^ |
| bv21-2__ccbv_32.12_ | 0,199 | 0,800 | 28,815 | 290 | < 10^-4^ |
| bv3-1__ccbv_18.1b_ | 0,291 | 0,826 | 32,878 | 629 | < 10^-4^ |
| bv3-2__ccbv_30.1_ | 0,242 | 0,826 | 38,659 | 638 | < 10^-4^ |
| bv5-4__ccbv_23.3_ | 0,368 | 0,801 | 16,799 | 296 | < 10^-4^ |
| bv5-3__ccbv_9.5_ | 0,291 | 0,814 | 26,206 | 425 | < 10^-4^ |
| bv5-7__ccbv_33.4_ | 0,318 | 0,802 | 19,935 | 305 | < 10^-4^ |
| bv6-5__ccbv_29.13_ | 0,095 | 0,797 | 45,035 | 269 | < 10^-4^ |
| bv6-1__ccbv_29.2_ | 0,127 | 0,806 | 42,661 | 338 | < 10^-4^ |
| bv6-12__ccbv_28.15_ | 0,097 | 0,796 | 44,373 | 266 | < 10^-4^ |
| bv23-3__ccbv_28.13_ | 0,198 | 0,793 | 26,687 | 242 | < 10^-4^ |
| bv6-18__ccbv_32.8b_ | 0,150 | 0,797 | 33,773 | 269 | < 10^-4^ |
| bv6-17__ccbv_32.14_ | 0,295 | 0,806 | 22,802 | 341 | < 10^-4^ |
| bv6-23__ccbv_32.23_ | 0,212 | 0,800 | 27,705 | 293 | < 10^-4^ |
| bv6-19__ccbv_32.16_ | 0,137 | 0,796 | 35,695 | 266 | < 10^-4^ |
| bv6-24__ccbv_35.1a_ | 0,218 | 0,793 | 24,378 | 245 | < 10^-4^ |
| bv6-25__ccbv_18.9b_ | 0,140 | 0,799 | 36,342 | 281 | < 10^-4^ |
| bv6-26__ccbv_18.11_ | 0,110 | 0,797 | 41,173 | 269 | < 10^-4^ |
| bv6-27__ccbv_16.6_ | 0,221 | 0,794 | 24,329 | 248 | < 10^-4^ |
| bv7-1bis__ccbv_22.1bis_ | 0,413 | 0,814 | 17,907 | 422 | < 10^-4^ |
| bv7-5__ccbv_36.1_ | 0,265 | 0,815 | 28,832 | 431 | < 10^-4^ |
| bv8-5__ccbv_27.3_ | 0,125 | 0,815 | 49,415 | 431 | < 10^-4^ |
| bv8-3__ccbv_28.6_ | 0,132 | 0,813 | 46,174 | 407 | < 10^-4^ |
| bv8-8__ccbv_32.19_ | 0,184 | 0,813 | 37,409 | 413 | < 10^-4^ |
| bv8-6like__ccbv15.4blike_ | 0,241 | 0,813 | 30,090 | 407 | < 10^-4^ |
| bv8-11__ccbv_16.3_ | 0,168 | 0,811 | 38,544 | 392 | < 10^-4^ |
| bv8-12__ccbv_12.3b_ | 0,129 | 0,815 | 49,380 | 443 | < 10^-4^ |
| bv9-5__ccbv_28.10_ | 0,209 | 0,812 | 33,437 | 404 | < 10^-4^ |
| bv9-6__ccbv_28.18_ | 0,105 | 0,805 | 47,971 | 329 | < 10^-4^ |
| ccbv_12.1 | 0,291 | 0,792 | 19,079 | 239 | < 10^-4^ |
| ccbv_18.13 | 0,155 | 0,814 | 42,639 | 422 | < 10^-4^ |
| ccbv_24.2 | 0,227 | 0,829 | 42,748 | 701 | < 10^-4^ |
| ccbv_32.18 | 0,145 | 0,795 | 33,481 | 257 | < 10^-4^ |
| ccbv_32.6 | 0,177 | 0,830 | 53,223 | 752 | < 10^-4^ |
| ccbv_32.7b | 0,212 | 0,799 | 27,082 | 281 | < 10^-4^ |
| ccbv_32.9b | 0,176 | 0,816 | 40,419 | 455 | < 10^-4^ |
| ccbv_28.4 | 0,220 | 0,808 | 30,560 | 362 | < 10^-4^ |
| crp4__ccbv_35.2_ | 0,217 | 0,813 | 33,045 | 407 | < 10^-4^ |
| crp3__ccbv_32.3_ | 0,195 | 0,803 | 30,809 | 314 | < 10^-4^ |
| ccv1__ccbv_13.3_ | 0,323 | 0,839 | 40,012 | 1094 | < 10^-4^ |
| ccbv_31.11 | 0,307 | 0,829 | 33,202 | 710 | < 10^-4^ |
| ccbv_31.12 | 0,310 | 0,829 | 33,333 | 722 | < 10^-4^ |
| bv11-3__ccbv_2.2_ | 0,264 | 0,836 | 44,847 | 962 | < 10^-4^ |
| bv9-7__ccbv_28.21_ | 0,165 | 0,813 | 39,866 | 407 | < 10^-4^ |
| bv6-8__ccbv_29.21_ | 0,138 | 0,799 | 36,437 | 281 | < 10^-4^ |
| bv9-3__ccbv_29.20_ | 0,285 | 0,811 | 25,445 | 389 | < 10^-4^ |
| bv8-14__ccbv_14.8_ | 0,198 | 0,812 | 34,768 | 398 | < 10^-4^ |
| cyst1__ccbv_19.6_ | 0,372 | 0,813 | 20,215 | 416 | < 10^-4^ |
| ep1-like6__ccbv_28.1_ | 0,332 | 0,830 | 31,598 | 737 | < 10^-4^ |
| ep1__ccbv_8.2_ | 0,361 | 0,832 | 30,582 | 818 | < 10^-4^ |
| ep1-like4__ccbv_5.5_ | 0,296 | 0,831 | 35,634 | 758 | < 10^-4^ |
| ep1-like5__ccbv_7.5_ | 0,409 | 0,811 | 17,344 | 389 | < 10^-4^ |
| ep1-like3__ccbv_1.5_ | 0,280 | 0,830 | 37,005 | 737 | < 10^-4^ |
| ep2__ccbv_2.4_ | 0,380 | 0,834 | 29,923 | 875 | < 10^-4^ |
| histone__ccbv_7.3_ | 0,156 | 0,810 | 39,920 | 380 | < 10^-4^ |
| ccv3__ccbv_13.5_ | 0,187 | 0,818 | 40,096 | 482 | < 10^-4^ |
| p94-like1__ccbv_7.1b_ | 0,387 | 0,842 | 37,815 | 1391 | < 10^-4^ |
| p94-like2__ccbv_7.2b_ | 0,158 | 0,838 | 69,464 | 1061 | < 10^-4^ |
| ptpa__ccbv_26.6_ | 0,179 | 0,836 | 60,552 | 977 | < 10^-4^ |
| ptpalpha__ccbv_17.4_ | 0,105 | 0,835 | 81,911 | 908 | < 10^-4^ |
| ptpb__ccbv_1.1_ | 0,204 | 0,830 | 47,180 | 734 | < 10^-4^ |
| ptpdelta__ccbv_26.1_ | 0,188 | 0,836 | 58,104 | 965 | < 10^-4^ |
| ptpe__ccbv_10.1_ | 0,167 | 0,835 | 61,249 | 908 | < 10^-4^ |
| ptph__ccbv_4.2_ | 0,180 | 0,836 | 59,774 | 959 | < 10^-4^ |
| ptpi__ccbv_1.2_ | 0,222 | 0,824 | 39,708 | 599 | < 10^-4^ |
| ptpk__ccbv_1.6_ | 0,190 | 0,831 | 51,418 | 776 | < 10^-4^ |
| ptpl__ccbv_1.7_ | 0,260 | 0,834 | 43,272 | 881 | < 10^-4^ |
| ptpn__ccbv_10.6_ | 0,199 | 0,836 | 55,041 | 947 | < 10^-4^ |
| ptpo__ccbv_4.3_ | 0,242 | 0,832 | 43,894 | 806 | < 10^-4^ |
| ptpq__ccbv_1.10_ | 0,246 | 0,825 | 37,158 | 611 | < 10^-4^ |
| ptpr__ccbv_7.4_ | 0,130 | 0,836 | 74,184 | 962 | < 10^-4^ |
| ptpt__ccbv_10.3_ | 0,217 | 0,808 | 30,707 | 362 | < 10^-4^ |
| ptpu__ccbv_14.1_ | 0,202 | 0,833 | 50,788 | 827 | < 10^-4^ |
| ptpv__ccbv_14.4_ | 0,145 | 0,835 | 67,384 | 917 | < 10^-4^ |
| ptpy__ccbv_17.2_ | 0,212 | 0,835 | 51,693 | 908 | < 10^-4^ |
| ptpz__ccbv_17.3_ | 0,156 | 0,833 | 61,215 | 833 | < 10^-4^ |
| rnaset2-like1__ccbv_23.4_ | 0,254 | 0,843 | 59,942 | 1583 | < 10^-4^ |
| rnaset2-like3__ccbv_25.6b_ | 0,209 | 0,837 | 54,935 | 1010 | < 10^-4^ |
| ser-rich2__ccbv_29.7_ | 0,268 | 0,812 | 27,009 | 395 | < 10^-4^ |
| ser-rich5__ccbv_28.14_ | 0,150 | 0,816 | 44,905 | 449 | < 10^-4^ |
| ser-rich8__ccbv_18.10_ | 0,122 | 0,796 | 37,997 | 263 | < 10^-4^ |

^a^ Statistical significance of the difference between observed and expected saturation indexes
