# Supplementary material for: Adaptive Selection on Bracovirus Genomes Drives the Specialization of Cotesia Parasitoid Wasps
Source: PLoS One. 2013 May 28;8(5):e64432. doi: 10.1371/journal.pone.0064432 (PMC3665748; doi:10.1371/journal.pone.0064432)
Supplement: Table S7 — Annotation of the 54 orthologs shared by the 4 Cotesia bracoviruses. (DOCX) [file pone.0064432.s007.docx]

Table S7: Annotation of the 54 orthologs shared by the 4 *Cotesia* bracoviruses

| **CskBV** | | **CsmBV** | | **F** | | **CcBV** | | **Function** | **WolfPSORT** |
| --- | --- | --- | --- | --- | --- | --- | --- | --- | --- |
| **Gene Name** | **Circle/Segment** | **Gene Name** | **Circle/Segment** | **Gene Name** | **Circle/Segment** | **Gene Name** | **Circle/Segment** |  |  |
| (CskBV_13.3)_(CskBV_13.3) | CskBV13 | (CsmBV_13.3)_(CsmBV_13.3) | CsmBV13 | gb\|AEE09595.1\| | CvBV29 | CcBV_18.2 | CcBV18 | unknown | extracellular |
| (CskBV_13.7)_(CskBV_13.7) | CskBV13 | (CsmBV_13.7)_(CsmBV_13.7) | CsmBV13 | gb\|AEE09591.1\| | CvBV29 | CcBV_13.2 | CcBV13 | unknown | nd |
| ank6-like_(CskBV_26.5) | CskBV26 | ank6-like_(CsmBV_26.5) | CsmBV26 | gb\|AEE09523.1\| | CvBV14 | ank-6_(CcBV_26.3b) | CcBV26 | viral ankyrin | extracellular |
| ank7-like_(CskBV_16.1) | CskBV16 | ank7-like_(CsmBV_16.1) | CsmBV16 | gb\|AEE09536.1\| | CvBV17 | ank-7_(CcBV_16.1) | CcBV16 | viral ankyrin | cytoplasm |
| ank-8_(CskBV_16.2) | CskBV16 | ank-8_(CsmBV_16.2) | CsmBV16 | gb\|AEE09535.1\| | CvBV17 | ank-8_(CcBV_16.2) | CcBV16 | viral ankyrin | cytoplasm |
| ben_(CskBV_27.2) | CskBV27 | ben_(CsmBV_27.2) | CsmBV27 | gb\|AEE09584.1\| | CvBV27 | ben-2_(CcBV_3.4) | CcBV3 | ben-domain protein | nucleus |
| ben_(CskBV_27.3) | CskBV27 | ben_(CsmBV_27.3) | CsmBV27 | gb\|AEE09583.1\| | CvBV27 | ben-14_(CcBV_24.1) | CcBV24 | ben-domain protein | cytoplasm |
| bv11_(CskBV_36.5) | CskBV36 | bv11_(CsmBV_36.5) | CsmBV36 | gb\|AEE09603.1\| | CvBV34 | bv11-4_(CcBV_36.3) | CcBV36 | unknown bv11 protein | nd |
| bv12-like_(CskBV_17.2) | CskBV17 | bv12-like_(CsmBV_17.2) | CsmBV17 | gb\|AEE09461.1\| | CvBV4 | bv12-2_(CcBV_10.5b) | CcBV10 | unknown bv12 protein | cytoskeleton |
| bv14_(CskBV_13.4) | CskBV13 | bv14_(CsmBV_13.4) | CsmBV13 | \|gb\|AEE09596.1\| | CvBV29 | bv14-2_(CcBV_18.3) | CcBV18 | unknown bv14 protein | extracellular |
| bv15_(CskBV_2.4) | CskBV2 | bv15_(CsmBV_2.4) | CsmBV2 | gb\|AEE09479.1\| | CvBV7 | bv15-2_(CcBV_2.5) | CcBV2 | unknown bv15 protein | extracellular |
| bv18_(CskBV_24.1) | CskBV24 | bv18_(CsmBV_24.1) | CsmBV24 | gb\|AEE09553.1\| | CvBV21 | bv18-2_(CcBV_27.6) | CcBV27 | unknown bv18 protein | mito |
| bv19_(CskBV_13.8) | CskBV13 | bv19_(CsmBV_13.8) | CsmBV13 | gb\|AEE09592.1\| | CvBV29 | bv19.2_(CcBV_13.1b) | CcBV13 | unknown bv19 protein | plas |
| bv2_(CskBV_2.5) | CskBV2 | bv2_(CsmBV_2.5) | CsmBV2 | gb\|AEE09483.1\| | CvBV7 | bv2-7_(CcBV_31.4) | CcBV31 | unknown bv2 protein | extracellular |
| bv21_(CskBV_32.1) | CskBV32 | bv21_(CsmBV_32.1) | CsmBV32 | gb\|AEE09565.1\| | CvBV24 | bv21-2_(CcBV_32.12) | CcBV32 | unknown bv21 protein | nucleus |
| bv5_(CskBV_33.1) | CskBV33 | bv5_(CsmBV_33.1) | CsmBV33 | gb\|AEE09451.1\| | CvBV1 | bv5-3_(CcBV_9.5) | CcBV9 | unknown bv5 protein | extracellular |
| bv6_(CskBV_28.10) | CskBV28 | bv6_(CsmBV_28.10) | CsmBV28 | gb\|AEE09544.1\| | CvBV20 | bv6-5_(CcBV_29.13) | CcBV29 | unknown bv6 protein | extracellular |
| bv6_(CskBV_32.14) | CskBV32 | bv6_(CsmBV_32.14) | CsmBV32 | gb\|AEE09574.1\| | CvBV24 | bv6-17_(CcBV_32.14) | CcBV32 | unknown bv6 protein | extracellular |
| bv6_(CskBV_32.9) | CskBV32 | bv6_(CsmBV_32.9) | CsmBV32 | gb\|AEE09569.1\| | CvBV24 | bv6-19_(CcBV_32.16) | CcBV32 | unknown bv6 protein | nd |
| bv6_(CskBV_35.2) | CskBV35 | bv6_(CsmBV_35.2) | CsmBV35 | gb\|AEE09460.1\| | CvBV3 | bv6-24_(CcBV_35.1a) | CcBV35 | unknown bv6 protein | nd |
| bv6-25-like_(CskBV_18.2) | CskBV18 | bv6-25-like_(CsmBV_18.2) | CsmBV18 | gb\|AEE09576.1\| | CvBV24 | bv6-25_(CcBV_18.9b) | CcBV18 | unknown bv6 protein | extracellular |
| bv6-26-like_(CskBV_18.1) | CskBV18 | bv6-26-like_(CsmBV_18.1) | CsmBV18 | gb\|AEE09575.1\| | CvBV24 | bv6-26_(CcBV_18.11) | CcBV18 | unknown bv6 protein | nd |
| bv8_(CskBV_28.14) | CskBV28 | bv8_(CsmBV_28.14) | CsmBV28 | gb\|AEE09572.1\| | CvBV24 | bv8-3_(CcBV_28.6) | CcBV28 | unknown bv8 protein | nd |
| bv8_(CskBV_35.1) | CskBV35 | bv8_(CsmBV_35.1) | CsmBV35 | gb\|AEE09459.1\| | CvBV3 | bv8-6like_(CcBV15.4blike) | CcBV15 | unknown bv8 protein | cytoplasm |
| bv8-like_(CskBV_16.3) | CskBV16 | bv8-like_(CsmBV_16.3) | CsmBV16 | gb\|AEE09537.1\| | CvBV17 | bv8-11_(CcBV_16.3) | CcBV16 | unknown bv8 protein | cytoskeleton |
| bv9_(CskBV_28.12) | CskBV28 | bv9_(CsmBV_28.12) | CsmBV28 | gb\|AEE09545.1\| | CvBV20 | bv9-5_(CcBV_28.10) | CcBV28 | unknown bv9 protein | cytoplasm |
| bv9_(CskBV_28.5) | CskBV28 | bv9_(CsmBV_28.5) | CsmBV28 | gb\|AEE09551.1\|a | CvBV20 | bv9-6_(CcBV_28.18) | CcBV28 | unknown bv9 protein | cytoplasm |
| CcBV_18.13-like_(CskBV_18.1) | CskBV18 | CcBV_18.13-like_(CsmBV_18.1) | CsmBV18 | gb\|AEE09518.1\| | CvBV13 | CcBV_18.13 | CcBV18 | unknown | mitochondrion |
| CcBV_24.2-like_(CskBV_24.4) | CskBV24 | CcBV_24.2-like_(CsmBV_24.4) | CsmBV24 | gb\|AEE09552.1\| | CvBV21 | CcBV_24.2 | CcBV24 | unknown | cytoplasm |
| CcBV_32.6-like_(CskBV_32.12) | CskBV32 | CcBV_32.6-like_(CsmBV_32.12) | CsmBV32 | gb\|AEE09571.1\| | CvBV24 | CcBV_32.6 | CcBV32 | unknown | cytoplasm |
| CcBV_32.7b-like_(CskBV_32.11) | CskBV32 | CcBV_32.7b-like_(CsmBV_32.11) | CsmBV32 | gb\|AEE09570.1\| | CvBV24 | CcBV_32.7b | CcBV32 | unknown | nucleus |
| crp3_(CskBV_32.13) | CskBV32 | crp3_(CsmBV_32.13) | CsmBV32 | gb\|AEE09573.1\| | CvBV24 | crp3_(CcBV_32.3) | CcBV32 | CRP3 | nd |
| crv1_(CskBV_13.5) | CskBV13 | crv1_(CsmBV_13.5) | CsmBV13 | gb\|AEE09597.1\| | CvBV29 | CcV1_(CcBV_13.3) | CcBV13 | CRV1 | extracellular |
| CskBV_2.2 | CskBV2 | CsmBV_2.2 | CsmBV2 | gb\|AEE09478.1\| | CvBV7 | CcBV_31.11 | CcBV31 | unknown | extracellular |
| CskBV_2.7 | CskBV2 | CsmBV_2.7 | CsmBV2 | gb\|AEE09481.1\| | CvBV7 | bv11-3_(CcBV_2.2) | CcBV31 | unknown | extracellular |
| CskBV_28.1 | CskBV28 | CsmBV_28.1 | CsmBV28 | gb\|AEE09549.1\| | CvBV20 | bv9-7_(CcBV_28.21) | CcBV28 | unknown | cytoplasm |
| CskBV_28.2 | CskBV28 | CsmBV_28.2 | CsmBV28 | gb\|AEE09550.1\| | CvBV20 | bv6-8_(CcBV_29.21) | CcBV29 | unknown | nd |
| ep2-like_(CskBV_13.1) | CskBV13 | ep1-like_(CsmBV_13.1) | CsmBV13 | gb\|AEE09594.1\| | CvBV29 | ep2-like2_(CcBV_13.4) | CcBV13 | EP2-like | extracellular |
| ep1-like_(CskBV_37.1) | CskBV37 | ep1-like_(CsmBV_37.1) | CsmBV37 | gb\|AEE09600.1\| | CvBV32 | ep1-like6_(CcBV_28.1) | CcBV28 | EP1-like | nd |
| ep2_(CskBV_2.1) | CskBV2 | ep2_(CsmBV_2.1) | CsmBV2 | gb\|AEE09482.1\| | CvBV7 | ep2_(CcBV_2.4) | CcBV2 | EP2 | extracellular |
| histone_(CskBV_7.1) | CskBV7 | histone_(CsmBV_7.1) | CsmBV7 | gb\|AEE09495.1\| | CvBV11 | histone_(CcBV_7.3) | CcBV7 | Viral Histone H4-like | cytoplasm-nucleus |
| lectin_(CskBV_13.9) | CskBV13 | lectin_(CsmBV_13.9) | CsmBV13 | gb\|AEE09593.1\| | CvBV29 | CcV3_(CcBV_13.5) | CcBV13 | Lectin-C | extracellular |
| p94_(CskBV_7.4) | CskBV7 | p94_(CsmBV_7.4) | CsmBV7 | gb\|AEE09498.1\| | CvBV11 | p94-like1_(CcBV_7.1b) | CcBV7 | P94-like | cytoplasm |
| p94-like_(CskBV_7.7) | CskBV7 | p94-like_(CsmBV_7.7) | CsmBV7 | gb\|AEE09494.1\| | CvBV11 | p94-like2_(CcBV_7.2b) | CcBV7 | P94-like | cytoplasm |
| ptp-a_(CskBV_26.1) | CskBV26 | ptp-a_(CsmBV_26.1) | CsmBV26 | gb\|AEE09524.1\| | CvBV14 | ptpa_(CcBV_26.6) | CcBV26 | PTP-A | cytoplasm |
| ptp-alpha_(CskBV_17.1) | CskBV17 | ptp-alpha_(CsmBV_17.1) | CsmBV17 | gb\|AEE09462.1\| | CvBV4 | ptpalpha_(CcBV_17.4) | CcBV17 | PTP-alpha | nucleus |
| ptp-delta_(CskBV_26.3) | CskBV26 | ptp-delta_(CsmBV_26.3) | CsmBV26 | gb\|AEE09519.1\| | CvBV14 | ptpdelta_(CcBV_26.1) | CcBV26 | PTP-delta | cytoplasm |
| ptp-e/x_(CskBV_17.5) | CskBV17 | ptp-e/x_(CsmBV_17.5) | CsmBV17 | gb\|AEE09465.1\| | CvBV4 | ptpe_(CcBV_10.1) | CcBV10 | PTP-EX | cytoplasm |
| ptp-h_(CskBV_4.2) | CskBV4 | ptp-h_(CsmBV_17.7) | CsmBV17 | gb\|AEE09489.1\| | CvBV9 | ptph_(CcBV_4.2) | CcBV4 | PTP-H | cytoplasm |
| ptp-r_(CskBV_7.2) | CskBV7 | ptp-r_(CsmBV_7.2) | CsmBV7 | gb\|AEE09496.1\| | CvBV11 | ptpr_(CcBV_7.4) | CcBV7 | PTP-R | cytoplasm-nucleus |
| ptp-thau(CskBV_17.6) | CskBV17 | ptp-thau(CsmBV_17.6) | CsmBV17 | gb\|AEE09467.1\| | CvBV4 | ptpt_(CcBV_10.3) | CcBV10 | PTP-thau | nucleus |
| ptp-Z_(CskBV_17.3) | CskBV17 | ptp-Z_(CsmBV_17.3) | CsmBV17 | gb\|AEE09477.1\| | CvBV6 | ptpz_(CcBV_17.3) | CcBV17 | ptp-Z | cytoplasm |
| ser-rich_(CskBV_28.8) | CskBV28 | ser-rich_(CsmBV_28.8) | CsmBV28 | gb\|AEE09551.1\|b | CvBV20 | ser-rich5_(CcBV_28.14) | CcBV28 | Ser-Rich protein | nucleus |
| ser-rich6-like_(CskBV_18.2) | CskBV18 | ser-rich6-like_(CsmBV_18.2) | CsmBV18 | gb\|AEE09516.1\| | CvBV13 | ser-rich8_(CcBV_18.10) | CcBV18 | Ser-Rich protein | nd |
