# Supplementary material for: Adaptive Selection on Bracovirus Genomes Drives the Specialization of Cotesia Parasitoid Wasps
Source: PLoS One. 2013 May 28;8(5):e64432. doi: 10.1371/journal.pone.0064432 (PMC3665748; doi:10.1371/journal.pone.0064432)
Supplement: File S1 — Comparative phylogenetic analyses of Cotesia and associated bracoviruses. Figure S1 Co-phylogeny between Cotesia wasps and their bracoviruses. (DOCX) [file pone.0064432.s008.docx]

Supporting Information

We estimated the global genetic divergence between the four PDVs by measuring the global amino acids (AA) identity between orthologous genes (see main text). The *Cotesia* bracoviruses displayed around 75% to 83% amino-acid identity between orthologous genes from different species. Nearly 97% amino-acid identity was found between the 2 CsBV strains issued from different geographic regions but belonging to the same wasp species (confirmed by allozyme markers [[1](#_ENREF_1)], microsatellite markers [[2](#_ENREF_2)], and reproductive compatibility [[3](#_ENREF_3)]). We used these genetic distances to reconstruct the bracovirus phylogeny in PHYLIP v3.6 [[4](#_ENREF_4)] using the Neighbor-Joining method and compared the tree to the wasp phylogeny. We used partial COI sequences of ten *Cotesia* wasps (*C. congregata*, *C. vestalis*, *C. glomerata*, *C. glabrata*, *C. melitaearum*, *C. koedelei*, *C. flavipes*, *C. sesamiae* Kitale, *C. sesamiae* Mombasa and *C. sesamiae* nonagrioides) and of *Glyptapanteles indiensis* (outgroup) to infer the wasp phylogeny. Sequences were retrieved from Genbank except for *C. sesamiae* sequences that were obtained by PCR using the LCO/HCO universal primers [[5](#_ENREF_5)] and were aligned with Mafft 6.8.11 [[6](#_ENREF_6)] then cured with BMGE [[7](#_ENREF_7)]. Maximum likelihood analysis (ML) was performed with PHYML program [[8](#_ENREF_8)] using the GTR substitution model, and support for node in ML tree was obtained from 10,000 nonparametric bootstrap iterations.

The comparison between both trees showed that bracovirus genome divergence mirrors the hymenopterans host phylogeny (Figure S1) as obtained previously using sequences of the *CrV1* gene [[9](#_ENREF_9)]. Co-speciation between the wasp and its integrated symbiotic virus is expected [[9](#_ENREF_9)]. The co-phylogeny we observe confirms the codiversification pattern between wasp and bracovirus at the whole bracovirus genome scale. However particular bracovirus genes might not follow this phylogeny in particular if they are implicated in adaptive processes and if their coalescence do not strictly follow speciation [[10](#_ENREF_10)].

Figure S1. Co-phylogeny between *Cotesia* wasps and their bracoviruses.

Left: parasitoid wasp maximum likelihood phylogeny based on COI sequences, support for nodes above branches indicates maximum likelihood nonparametric bootstraps (10,000 replicates); Right: bracovirus neighbor-joining tree based on pairwise amino-acid distance obtained from all orthologs shared by each pair of bracovirus genome.

**References**

1. Kimani-Njogu SW, Overholt WA, Woolley JB, Omwega CO (1998) Electrophoretic and phylogenetic analyses of selected allopatric populations of the Cotesia flavipes complex (Hymenoptera : Braconidae), parasitoids of cereal stemborers. Biochem Syst Ecol 26: 285-296.

2. Branca A (2009) Diversification écologique du parasitoïde africain Cotesia sesamiae: rôle des partenaires symbiotiques: Université Pierre et Marie Curie. 470 p.

3. Mochiah MB, Ngi-Song AJ, Overholt WA, Stouthamer R (2002) Variation in encapsulation sensitivity of Cotesia sesamiae biotypes to Busseola fusca. Entomol Exp Appl 105: 111-118.

4. Felsenstein J (2005) PHYLIP (Phylogeny Inference Package) version 3.6. In: author Dbt, editor. 3.6 ed. Seattle: Department of Genome Sciences, University of Washington.

5. Folmer O, Black M, Hoeh W, Lutz R, Vrijenhoek R (1994) DNA primers for amplification of mitochondrial cytochrome c oxidase subunit I from diverse metazoan invertebrates. Mol Mar Biol Biotechnol 3: 294-299.

6. Katoh K, Toh H (2008) Recent developments in the MAFFT multiple sequence alignment program. Brief Bioinform 9: 286-298.

7. Criscuolo A, Gribaldo S (2010) BMGE (Block Mapping and Gathering with Entropy): a new software for selection of phylogenetic informative regions from multiple sequence alignments. BMC Evol Biol 10: 210.

8. Guindon S, Lethiec F, Duroux P, Gascuel O (2005) PHYML Online--a web server for fast maximum likelihood-based phylogenetic inference. Nucleic Acids Res 33: W557-559.

9. Whitfield JB, Asgari SB (2003) Virus or not? Phylogenetics of polydnaviruses and their wasp carriers. J Insect Physiol 49: 397-405.

10. Degnan JH, Rosenberg NA (2009) Gene tree discordance, phylogenetic inference and the multispecies coalescent. Trends in Ecology &amp; Evolution 24: 332-340.
